# Patterns of Deep-Water Coral Diversity in the Caribbean Basin and Adjacent Southern Waters: An Approach based on Records from the R/V Pillsbury Expeditions

**DOI:** 10.1371/journal.pone.0092834

**Published:** 2014-03-26

**Authors:** Iván Hernández-Ávila

**Affiliations:** 1 Departamento de Ciencias, Unidad de Cursos Básicos, Núcleo de Nueva Esparta, Universidad de Oriente, Margarita Island, Venezuela; 2 IFREMER, Unité de recherche Étude des Écosystèmes Profonds, Laboratoire Environnement Profond, Plouzané, France; Bangor University, United Kingdom

## Abstract

The diversity of deep-water corals in the Caribbean Sea was studied using records from oceanographic expeditions performed by the R/V *Pillsbury*. Sampled stations were sorted according to broad depth ranges and ecoregions and were analyzed in terms of species accumulation curves, variance in the species composition and contributions to alpha, beta and gamma diversity. According to the analysis of species accumulation curves using the Chao2 estimator, more diversity occurs on the continental slope (200–2000 m depth) than on the upper continental shelf (60–200 m depth). In addition to the effect of depth sampling, differences in species composition related to depth ranges were detected. However, the differences between ecoregions are dependent on depth ranges, there were fewer differences among ecoregions on the continental slope than on the upper continental shelf. Indicator species for distinctness of ecoregions were, in general, Alcyonaria and Antipatharia for the upper continental shelf, but also the scleractinians *Madracis myriabilis* and *Cladocora debilis*. In the continental slope, the alcyonarian *Placogorgia* and the scleractinians *Stephanocyathus* and *Fungiacyathus* were important for the distinction of ecoregions. Beta diversity was the most important component of gamma diversity in the Caribbean Basin. The contribution of ecoregions to alpha, beta and gamma diversity differed with depth range. On the upper continental shelf, the Southern Caribbean ecoregion contributed substantially to all components of diversity. In contrast, the northern ecoregions contributed substantially to the diversity of the Continental Slope. Strategies for the conservation of deep-water coral diversity in the Caribbean Basin must consider the variation between ecoregions and depth ranges.

## Introduction

Deep-water corals represent a diverse group of cnidarians, including scleractinians, zoanthids, alcyonarians, antipatharians and hydrocorals (Stylasteridae), and are widely distributed in all oceans at depths below 50 m [1], [2]. Despite its diversity, this group is ecologically important because it includes many habitat-building species (e.g., scleractinian formations and octocoral aggregations) and many species form associations with other species occurring on their surfaces [3]. Coral formations represent one of the most important biotic habitats in the deep-water environment [4]. However, they are vulnerable to a range of anthropogenic threats that include trawl fisheries, oil exploitation, mining and ocean acidification [5]–[8].

The Caribbean Sea is a semi-enclosed basin of approximately 2.75⋅10^6^ km^2^ bounded by Central and South America and the arc of the Greater and Lesser Antilles. The average depth is approximately 2400 m, with around 75% of the seafloor located at depths corresponding to abyssal waters and the continental rise (below 1800 m) and the rest located on the continental shelf and continental slope. Hadal depths are uncommon and are restricted to the Cayman Trough and the Puerto Rico Trench. Sills and ridges separate the Caribbean into five basins: the Grenada, Venezuelan, Colombian and Yucatan Basins and the Cayman Trough [9]. According to Spalding *et al*. [10], the Caribbean Sea includes five marine ecoregions: the Eastern Caribbean in the Grenada Basin; the Southern Caribbean in the Venezuelan Basin; the Southwestern Caribbean in the Colombian Basin; the Western Caribbean in the Yucatan Basin; and the Greater Antilles formed by Cuba, Hispaniola, Puerto Rico and the Cayman Islands.

Topographic configuration of the Caribbean is very heterogeneous in terms of depth, slope and elevations, such as volcanoes and seamounts. For example, more seamounts are found in the Greater Antilles and the Yucatan Peninsula and mud volcanoes have been found in the Southern Caribbean [9], [11], [20]. In general, deep-water hydrography of the Caribbean consists in stratified masses of water in the upper 2000 m and unstratified water below this depth [12]. Below the mixed layer are found Subtropical Underwater (SUW 110–200 m), composed mainly of Southern SUW and Eastern Tropical Water, both entering through passages in the Lesser Antilles, and Northern SUW that inflows through the Windward Passage and the Anegada-Jungfern complex. Central Water (CW), composed mainly of eastern South Atlantic CW and secondarily of North Atlantic CW, follows a pattern of inflow similar to the SUW. Antarctic Intermediate Water (AAIW) is the main water mass between 800–1100 m and inflows mainly via the Grenada Passage. Deep inflow of North Atlantic Deep Water and eventual outflow occur between 1200–2000 m through the Windward Passage and the Anegada-Jungfern complex [13], [14]. Below 2000 m, elevated sills limit the interchange between the Caribbean and the Atlantic, although episodic ventilation could occur [14]. The Yucatan Peninsula serves as a gateway for most of the outflow water from the Caribbean [15]. Additionally, the flux of particulate organic carbon (POC flux) shows spatial differences in the Caribbean. Models [16], [17] estimate more POC flux to the deep benthos in the Southern Caribbean due to the influence of coastal upwelling and riverine inflow. Moreover, the Lesser Antilles could be affected by the Orinoco-Amazonas inflow [18], [19].

Deep-water corals in the Caribbean have been studied frequently since the 19^th^ century (see historical review in [20]). Although most of these studies have focused on taxonomy and systematics, several studies have described the large-scale distribution of deep-water corals in the Atlantic [21]–[23], [34] or at the meso- or local scale in certain regions of the Caribbean [24]–[26]. These studies highlight the Caribbean as a globally important center of diversity for deep-water corals [1]. Most of the margins of the Caribbean Basin offer suitable habitats for deep-water corals [27]–[29], and many habitat-building species are widespread, forming deep-water reefs and similar habitats [20], [24]. However, the general patterns of deep-water coral diversity in the Caribbean remain elusive, as is the case for most of the deep-water benthos due to fragmented and (apparently) insufficient sampling efforts [9].

Several models have been proposed to describe general patterns of deep-water diversity relative to depth and spatial distributions. In an analysis of marine diversity according to depth, Gray *et al*. [30] postulated that shallow and deep habitats support similar numbers of species in similar areas and that the overall diversity relative to the number of habitats and number of species per habitat must be higher in the shallow-water benthos than in the deep-water benthos. Other studies support the notion that there is higher species diversity in assemblages of the deep-water benthos, in relation to assemblages of the continental shelf, for particular taxonomic groups and for complete community assemblages [7], [31], [32]. For deep-water corals, Cairns [1] has shown that most species inhabit the depths of the continental slope, although the ratio of species inhabiting the shelf (depths of 50–200 m) to species inhabiting the slope is relatively high.

Many deep-water coral species are widely distributed, and models of habitat suitability usually include broad connections between regions [28], [29]. These patterns seem to indicate that the composition of deep-water corals could be homogeneous at a regional scale. For the Caribbean Sea, Miloslavich *et al*. [9] failed to detect regional differences in shallow-water faunal composition for five major taxonomic groups, including corals. This result suggests that the classification of marine ecoregions in the Caribbean did not apply to shallow-water benthic fauna. However, according to Levin *et al.* [32], deep-water species diversity shows geographic variation at a scale of 100–1000 km, the same scale as that of ecoregions in the Caribbean, and Briggs *et al.* [33] report regional differences in species composition in the abyssal depths of the Caribbean. Furthermore, the diversity contours of azooxanthellate Scleractinia presented in Cairns [1] and the cluster analysis in Cairns & Chapman [23] suggests that species are not homogeneously distributed in the Caribbean Basin. In contrast, Dawson [34] re-analyzed previous data [21]–[23] and did not find evidence of subdivision in regions within the Caribbean based on the distribution of species, excluding surrounding regions, although an effect of depth in species composition was detected. Therefore, there is an on-going debate on whether the spatial distribution of deep-water corals in the Caribbean region is heterogeneous [23] or homogeneous [34], which is very important to resolve as it will determine the most appropriate conservation strategy for this ecosystem.

The identification of the patterns of spatial distribution of the deep-water benthos on a large scale is limited by the costs and logistic challenges involved in studying these systems. Also, analyses of databases are usually difficult due to differences in the time of sample collection, methods, identification and coverage. Previous approaches related to distribution of deep-water corals in the area [23], [34] were based on ordination methods of data pooled into subunits, which offers an excellent framework but overlooks the within-unit variation of assemblages in order to detect differences among units. However, a sampling program specific to the Caribbean could provide the opportunity to conduct an analysis of general patterns.

**Figure 1:**
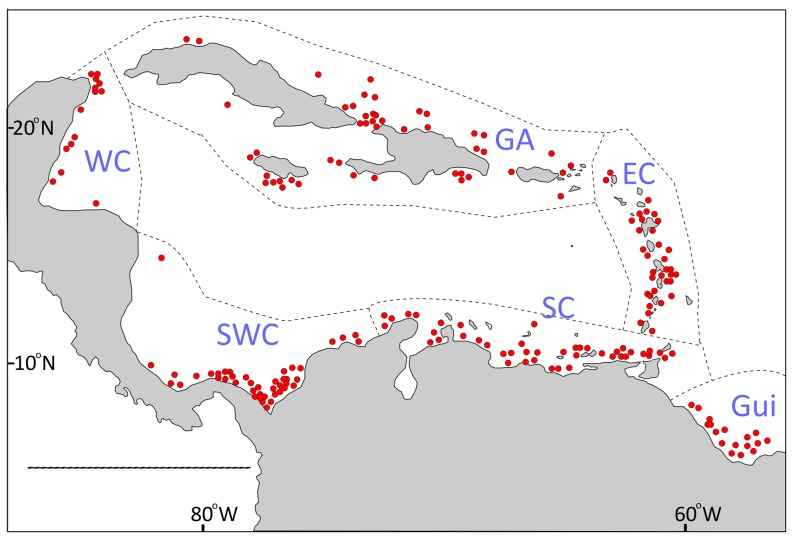
Stations of deep-water coral records analyzed in the present study. EC: Eastern Caribbean, Gui: Guianian, SC: Southern Caribbean, SWC: Southwestern Caribbean, WC: Western Caribbean. Regions adjusted from Spalding *et al*. [10].

An extensive sampling program in the Caribbean and adjacent waters was conducted by the R/V *Pillsbury* of the Rosenstiel School of Marine and Atmospheric Science (RSMAS), University of Miami. These samples were collected at approximately 800 stations in shallow- and deep-water habitats over a relatively short period (approximately 5 years). These expeditions produced records of corals below a depth of 60 m for 204 stations in the Caribbean Basin and Guiana (Fig. 1), and were assembled together in a database that provides a unique opportunity to overcome the problems stated above regarding the identification of spatial distribution patterns in the Caribbean. Although the dataset from the *Pillsbury* expedition does not comprise all species and records of deep-water corals in the Caribbean, the number of species and records and the distribution of stations suggest that the dataset could furnish a representative sample for determining general patterns of diversity. The aim of this paper is to determine the patterns of spatial distribution of deep-water coral diversity in the Caribbean in terms of wide depth ranges and ecoregions, including the adjacent Guianian ecoregion, based on the records of the *Pillsbury* expeditions.

## Materials and Methods

The database contains records of deep-water corals collected by the R/V *Pillsbury* from 1966 through 1971 in the Caribbean and the adjacent southern waters (Guianian region). The complete depth range covered between approximately 60 and 5400 m, representing 204 stations bordering almost the entire Caribbean basin (Fig. 1), except for part of the Central American region where the shelf is shallow. Samples were collected with 10-ft or 41-ft otter trawls. Specific information for each sample is found in Voss [35]–[38], Staiger [39]–[41], and Staiger & Voss [42]. Specimens were identified by major coral specialists (FM Bayer, SD Cairns, DM Opresko and collaborators) and deposited in the U.S. National Museum of Natural History (Smithsonian Institution-NMNH) and the Marine Invertebrate Museum (MIM) of RSMAS, University of Miami. Station and depth data related to the specimens deposited in the NMNH was obtained from the collection database (http://invertebrates.si.edu/collections.htm), and for the species held in MIM-RSMAS by examination of records. Taxonomic records were revised according to Williams and Cairns (http://researcharchive.calacademy.org/research/izg/OCTOCLASS.htm), the World Marine Species Database (www.marinespecies.org/) and taxonomic treatises [21], [22]. Any names not found in these sources were excluded from the analysis. The database was sorted by presence-absence of species by station, avoiding duplicate records among data sources.

The sampling stations were classified according to Spalding *et al.* [10] based on the following ecoregions: Greater Antilles (50 stations,st), Eastern Caribbean (35 st), Southern Caribbean (45 st), Southwestern Caribbean (43 st), Western Caribbean (14 st) and Guianian (17 st). These ecoregions comprise the border of the Caribbean Basin and its adjacent southern ecoregion, which were divided into sub-basins and defined by consensus of biogeographic studies and expert opinions [10]. This classification is similar to the regions proposed by Cairns & Chapman [23] for deep-water Scleractinia deduced by cluster analysis (except by division of the “insular” cluster [23]). Nevertheless, the northern coasts of Cuba, Hispaniola and Puerto Rico, assigned by Spalding *et al.* [10] to the Bahamian ecoregion, are considered part of the Greater Antilles for the purpose of this study. Depth ranges were separated according to McClain & Hardy [43] except for the inclusion of the upper continental shelf, ranging from 60 to 200 m (103 st.). Other depth ranges considered include the continental slope (200–2000 m- 93 st.), the continental rise (2000–4000 m- 5 st.) and the abyssal depths (4000–6000 m- 3 st.). The boundaries of the upper continental shelf and the continental slope in this classification coincide with the common criteria for defining shallow and deep corals habitats[1], [21]–[23], [34], [44] and the low occurrence of Scleractinia below 2000 m depth [1]. The other depth ranges were selected based on commonly accepted marine depth ranges [10].

To illustrate the patterns of spatial distribution of species relative to depth, the distribution range of each species was estimated according to the minimum and maximum depths recorded in this study. Moreover, for the upper continental shelf and the continental slope, species accumulation curves were plotted using two approaches: 1) the accumulation of species by sampling effort and 2) the Chao2 estimator [45], [46]. The Chao2 estimator [45] was selected for this analysis because it is a non-parametric approach that considers the frequency of each species in the samples. This method shows high precision for estimating simulated communities [47] and has been used for the analysis of the richness of marine fauna [47]–[49]. Although the Chao2 estimator is more conservative than some parametric methods [50], it allows the total species richness to be compared between depth ranges without parametric assumptions [51]. To avoid the bias resulting from the selection of only stations with positive records, the estimates of coral richness were derived from calculations that also included stations with negative coral records from the expedition but those that included other megafaunal invertebrates (e.g., echinoderms, gastropods, decapod crustaceans). Differences in species accumulation curves between depth ranges were studied by graphical comparison of their respective 95% confidence intervals [52], and by t-student test following Guerra-Castro [53].

The null hypothesis of no differences among depth ranges and regions was tested using a permutational multivariate analysis of variance (PERMANOVA, [54]) on a matrix of Sorensen's similarity index for each pair of samples. The depth of each sample was included as a covariable in the analysis. Variation among samples within the same ecoregion and depth range was used to generate the residuals for the model. The distances between samples according to their similarity were represented in a non-metric multidimensional scaling plot (MDS). The factor Depth Range had only two levels: upper continental shelf and continental slope. Records for the continental rise and abyssal ranges were scarce and did not allow comparisons. Additionally, permutational analysis of multivariate dispersions (PERMDISP) [55] and pairwise PERMANOVA tests between regions were performed for each depth range. Frequent (i.e., indicator) species contributing to differences between ecoregions and depth ranges were identified using an information statistic (*I*-test, 2ΔI) [56].

The relative contribution of the regions to the overall diversity was estimated based on the additive partitioning of diversity according to Lu *et al*. [57]. For this purpose, species lists were pooled by ecoregion, grouped according to the pairwise test for the upper continental shelf and for the continental slope (except for distant ecoregions), and depth range. Gamma diversity, representing the total richness recorded in the present study, was estimated as an additive partitioning of alpha and beta diversity: 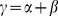.The overall α diversity, the richness for each ecoregion and depth combination, was expressed as 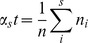, where *n* is the number of ecoregion-depth pairs, and *n*_i_ the number of ecoregions with records of the *i* ^th^ species. The partitioning of α for the *k* ^th^ ecoregion-depth combination was calculated as 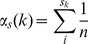, where *s_k_* represents each record for the ecoregion-depth combination. Similarly, beta diversity was calculated with 
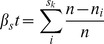
, partitioned using 
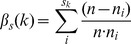
, and gamma diversity with 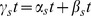, partitioned using 
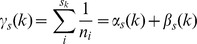
. The components of diversity were estimated for each ecoregion to evaluate their contribution to gamma diversity. In this case, this approach was not used to estimate the overall diversity, but to estimate the relative importance of alpha and beta diversity and the relative contribution of each ecoregion-depth combination. An estimate of between ecoregion-depth differentiation was calculated as 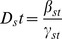. A value of D_st_>0.5 means that most diversity is distributed among units (ecoregion-depth combinations) [57]. After estimating the components of diversity partitioned by ecoregion, they were expressed as relative contribution to the overall component: 
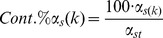
; 
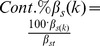
; 
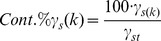
.

In certain cases, taxa were identified only to the generic level. In these cases, the taxon was assumed to differ from other species identified in the same genus. However, if identification at the generic level corresponded to a species previously recorded in the database, the partitioning of records could affect the data analysis. To estimate if this effect influenced the results, all data were aggregated to the generic level, and certain data analyses, including taxa accumulation according to sampling effort, the PERMANOVA test, the pairwise test and the MDS plot, were repeated and compared with the analysis based on identification to the species level. In addition, a RELATE analysis [55] was performed to compare the matrices of resemblance between samples based on identification to the species level and to the generic level. The results of these analyses are shown in the Tables S1 in File S1 and Table S2, Fig. S1–S2 in File S2.

## Results

A total of 218 species were recorded, belonging to almost all major groups of deep-water corals (105 Alcyonacea, 38 Antipatharia, 73 Scleractinia, 2 Anthoathecatae). The dataset includes 935 records from 204 stations and both colonial and solitary coral species. Alcyonarians, antipatharians and azooxanthellate scleractinians comprised most of the species, but several zooxanthellate and aposymbiotic species were recorded, especially from the upper continental shelf (Table S3 in File S2). Although some taxa have few records and species compared with previous records for the Caribbean Basin, such as Stylasteridae [58], [59], in general the dataset is a representative sample of the deep-water coral diversity of the area.

**Figure 2:**
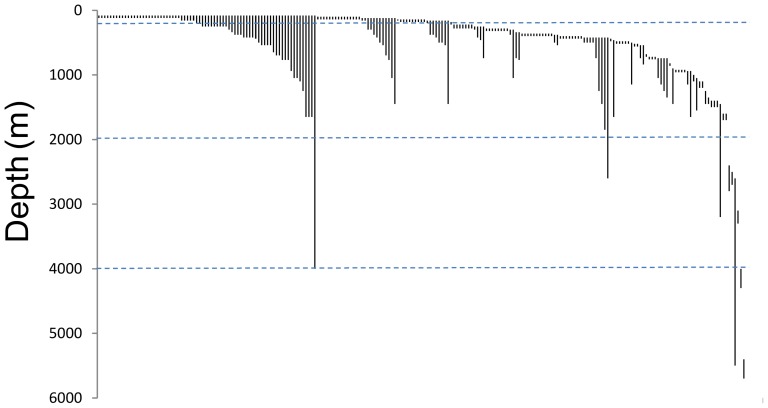
Depth intervals of species according to sample. Dashed lines represent limits of depth ranges.

Most of the species were found at depth intervals that included the continental slope. The next most common group consisted of the species found on the upper continental shelf (Fig. 2). In all, 58.2% of the species whose depth intervals included the upper continental shelf were also present below this depth range. A total of 62.9% of the species whose depth intervals included the continental slope were restricted to this depth range. More species (151 species) were found on the continental slope than on the upper continental shelf (123 species). The continental rise and abyssal ranges harbored few species (7 and 4 species, respectively), but the low sampling effort in these ranges precluded any comparative approach.

**Figure 3:**
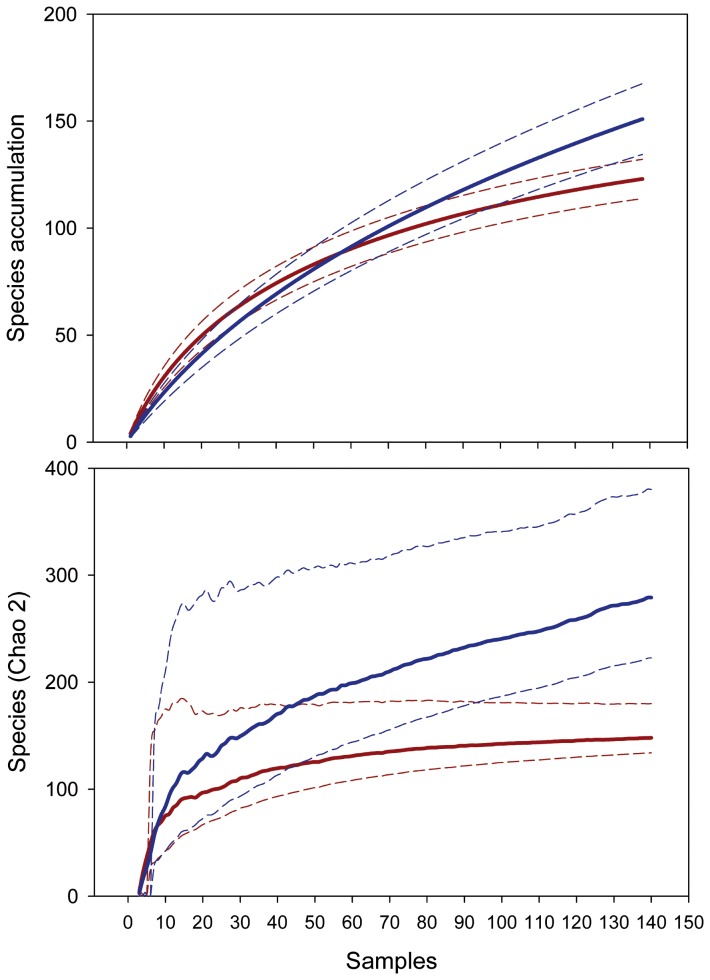
Accumulative species curves (S obs and Chao 2) for the upper continental shelf (red) and the continental slope (blue), dashed lines represent 95% confidence intervals.

The species accumulation curve of the upper continental shelf estimated a richness of 123±4.69 species (mean ± sd) (n = 138), and 151±8.44 species for the continental slope (n = 138) (t_999_ = 91.65, p<0.0001). The Chao2 estimator for species on the upper continental shelf was 148.02±10.98 species, slightly more than one-half of the species predicted for the continental slope (279.18±38.81 species) (t_999_ = 102.09, p<0.0001). The shape of the accumulation curve for the continental slope suggested that the species estimate could increase with more sampling. For either estimator, the 95% confidence intervals of the curves of upper continental shelf and continental slope did not overlap at the end of the curves (Fig. 3).

**Figure 4:**
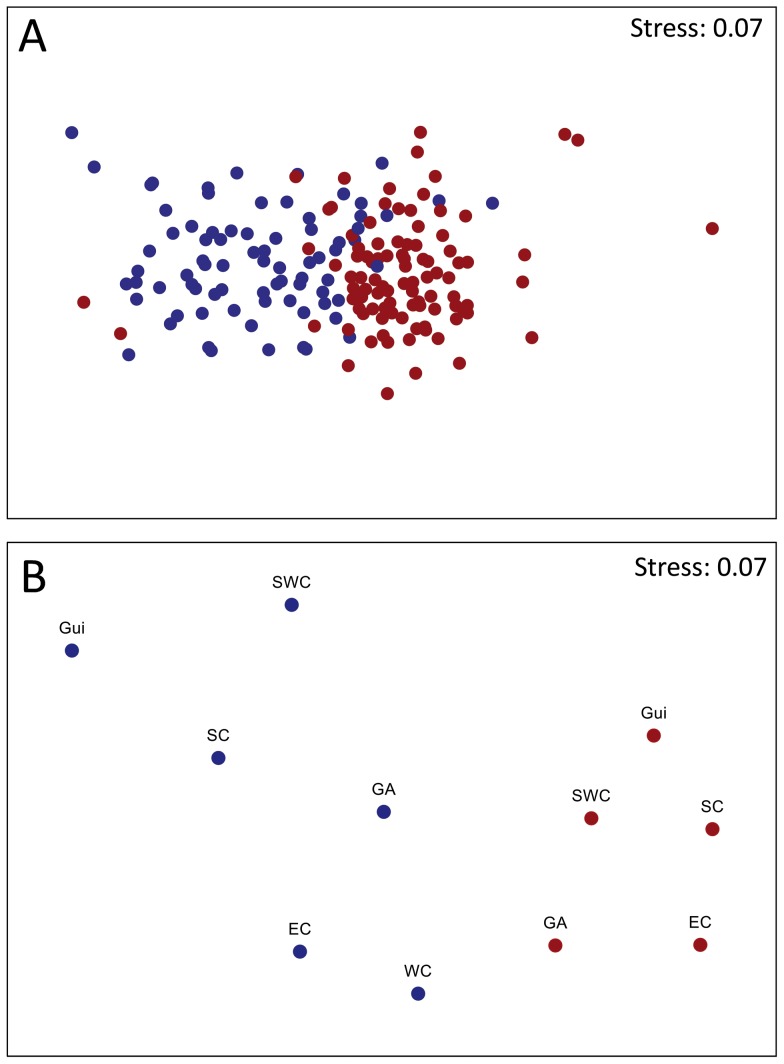
A. nm-MDS of the similarity between stations, estimated using Sorensen's similarity index, according to depth range. B. nm-MDS based on distance between centroids of ecoregions. Red, upper continental shelf; blue, continental slope. GA: Greater Antilles, EC: Eastern Caribbean, WC: Western Caribbean, Gui: Guianian, SC: Southern Caribbean, SWC: Southwestern Caribbean. 4A, 12 outliers were excluded. 4B, WC from the upper continental shelf excluded due lack of samples.

The PERMANOVA test based on Sorensen's similarity index between samples indicated significant differences in coral assemblages related to the depth of the sample, depth ranges and ecoregions (Table 1). A significant interaction was found between depth ranges and ecoregions, which suggest that ecoregional patterns depended on depth ranges. Differences between the composition of the upper continental shelf and continental slope species were detected in all ecoregions (pairwise test, p<0.001 in each case) except for the Western Caribbean due to a lack of samples from the upper continental shelf. A non-metric MDS based on the resemblance among all stations produced a degenerate solution (an accumulation of rankings of similarities in extreme positions of the plot). This outcome was associated with the occurrence of few stations that did not share their species composition with any other station due to the extreme rarity of the species (e.g., stations with only one species that did not occur in the other samples had a similarity of 0). These cases represented approximately 6.4% of the studied stations and 1.6% of the species records. A non-metric MDS plot excluding these stations showed differences in composition between the upper continental shelf and the continental slope (Fig. 4A). *I*-tests between depth ranges showed that indicator species of the upper continental shelf were mainly alcyonarians and antipatharians, but for the continental slope, most indicator species belonged to order Scleractinia (Table 2). Although the continental slope had higher species richness, fewer indicator species were found there than on the upper continental shelf, suggesting that differences were sustained in part by infrequent (rare) species. The continental slope showed more rare species (i.e., single record) than the upper continental shelf (χ^2^ = 25.37, p<0.001, df = 1) although sampling efforts were similar.

**Table 1:** PERMANOVA for similarity in species composition of deep-water corals (Sorensen). Bold denote significant differences.

Source	df	SS	MS	Pseudo-F	P(perm)	perms
Depth (cov)	1	59802	59802	14.661	**0.0001**	9843
Depth Range: De	1	23590	23590	5.783	**0.0001**	9874
Ecoregion: Er	5	43290	8657.9	2.123	**0.0001**	9725
De × Er	5	34139	6827.8	1.674	**0.0001**	9692
Residuals	180	734230	4079.1			
Total	192	895060				

**Table 2:** Indicator species for each depth range as determined using the *I*-test.

Species	UCS (101)	CS (92)	2ΔI
*Acanella* sp.	0	26	18.68
*Diodogorgia* sp.	27	0	16.92
*Ellisella* sp.	31	1	16.41
*Stephanocyathus diadema*	0	19	13.22
*Fungiacyathus symmetricus*	0	13	8.82
*Bellonella rubistella*	14	0	8.31
*Antipathes lenta*	24	3	8.27
*Deltocyathus eccentricus*	0	11	7.4
*Deltocyathus italicus*	0	10	6.7
*Leptogorgia* sp.	11	0	6.45
*Swiftia excerta*	11	0	6.45
*Tanacetipathes barbadensis*	10	0	5.84
*Balanophyllia dineta*	10	0	5.84
*Nicella goreaui*	9	0	5.24
*Nicella guadalupensis*	9	0	5.24
*Scleracis* sp.	15	2	4.74
*Telesto* sp.	12	1	4.59
*Cladocora debilis*	7	0	4.04
*Stephanocyathus coronatus*	0	6	3.95
*Stephanocyathus paliferus*	0	6	3.95

Occurrence in samples of upper continental shelf (UCS) samples and in continental slope (CS) samples are given. The total sample size is given in parentheses. Only species with 2ΔI>3.84 are shown.

**Figure 5:**
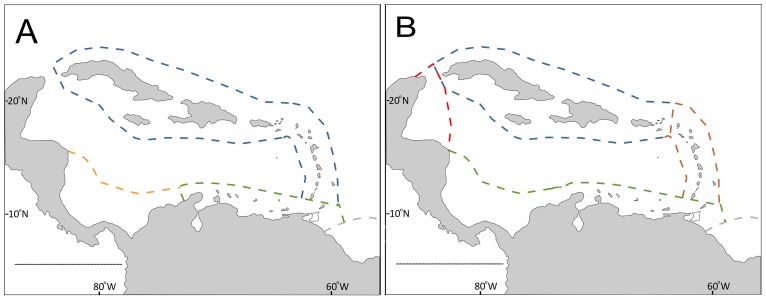
Interpretation of pairwise analysis between ecoregions by depth ranges. (A) Upper continental shelf, (B) continental slope. Affinities with the Guianian ecoregion were not resolved (see Results) and affinities between the Greater Antilles and Southern ecoregions for the continental slope are not shown.

According to the *a posteriori* test, the similarity among ecoregions changed according to depth range (Table 3, Fig. 4B). For the upper continental shelf, differences were not related to changes in multivariate dispersions between ecoregions (PERMDISP, F = 1.10, p = 0.90, df_d_ = 95), which support the detection of species turnover between ecoregions. Differences in assemblage composition were found in all ecoregions except for the Greater Antilles-Eastern Caribbean group, and the Guianian ecoregion, which showed p-values that did not allow the assumption of differences or similarities. Ecoregions on the continental slope showed differences in multivariate dispersions (PERMDISP F = 4.138, p = 0.431, df_d_ = 86) due to low dispersion of the Guianian ecoregion. Excluding the former ecoregion, the multivariate dispersions were not different (PERMDISP F = 2.439, p = 0.125, df_d_ = 82). The Greater Antilles was different from the Eastern Caribbean and Western Caribbean. These latter two ecoregions also were different from the Southern Caribbean-Southwestern Caribbean group (Fig. 5). However, no differences were found between the Greater Antilles and Southern ecoregions, that shown an overlap of assemblages between separate regions.

**Table 3:** Type I error probabilities for pairwise *t*-tests on the similarity in deep-water coral species composition between ecoregions (columns vs. rows).

Upper continental shelf					
	GA	EC	Gui	SC	SWC
EC	0.1322				
Gui	**0.0009**	0.0330			
SC	**0.0001**	**0.0001**	0.0379		
SWC	**0.0001**	**0.0019**	0.0215	**0.0001**	
WC	0.3366	0.1142	0.2547	0.1532	0.2714

Continental slope					
	GA	EC	WC	SWC	SC
EC	**0**.**0072**				
WC	0.0143*	0.1627			
SWC	0.0429	**0**.**0001**	**0**.**0015**		
SC	0.1804	**0**.**0032**	**0**.**0016**	0.1302	
Gui+	0.0757	**0**.**0011**	**0**.**0063**	0.1780	0.6011

GA: Greater Antilles, EC: Eastern Caribbean, Gui: Guianian, SC: Southern Caribbean, SWC: Southwestern Caribbean, WC: Western Caribbean. Significant differences are shown in bold. Gray denote low sampling effort.

^*^Marginal p-value.

+ PERDISM p<0.05.

Differences among ecoregions were explained in part by changes in frequency of occurrence of coral species as indicated by the *I*-test (Table 4). For the upper continental shelf, the Greater Antilles-Eastern Caribbean group differs from others ecoregions because it has higher frequencies of *Madracis myriaster* and *Nicella guadalupensis*. The Southern Caribbean ecoregion showed high frequency of the alcyonarians *Bellonella rubistella, Aphanipathes pedata* and *Thesea* sp. and the scleractinians *Balanophyllia dineta* and *Cladocora debilis*. The Southwestern Caribbean ecoregion showed a higher frequency of *Diodogorgia* sp. than the Greater Antilles. The Guianian did not show indicator species, which is consistent with the pairwise analysis. For the continental slope, the scleractinians were more important to determine the differences between ecoregions. In the Greater Antilles ecoregion, *Stephanocyathus diadema* was important, and was also frequent in the Southern Caribbean-Southwestern Caribbean along with *Stephanocyathus paliferus*. Higher frequency of *Fungiacyathus symetricus* was important for differences in the Eastern Caribbean ecoregion. The Western Caribbean ecoregion had no species that showed more frequencies than in other ecoregions.

**Table 4:** Indicator species in each ecoregion detected using the *I*-test.

	Upper continental shelf			
	GA+EC	SC	SWC	Gui
**GA+EC**		***Bellonella rubistella***	*Diodogorgia* sp.	
		*Balanophyllia dineta*		
		*Leptogorgia* sp.		
		*Aphanipathes pedata*		
		*Cladocora debilis*		
**SC**	*Madracis myriaster*			
**SWC**	***Madracis myriaster***	*Thesea sp.*		
	*Nicella guadalupensis*	*Balanophyllia dineta*		
		*Aphanipathes pedata*		
		*Cladocora debilis*		
		*Bellonella rubistella*		
**Gui**	*Madracis myriaster*	*Bellonella rubistella*		
		*Thesea* sp.		
	**Continental slope**			
	**GA**	**EC**	**SWC+SC**	**WC**
**GA**		*Fungiacyathus symmetricus*	*Stephanocyathus paliferus*	
**EC**	*Stephanocyathus diadema*		***Stephanocyathus diadema***	
**WSC+SC**		***Fungiacyathus symmetricus***		
**WC**			*Stephanocyathus diadema*	

Ecoregions were grouped according to pairwise analysis. GA: Greater Antilles, EC: Eastern Caribbean: WS: Western Caribbean, SWC: Southwestern Caribbean, SC: Southern Caribbean, Gui: Guianian.

Pairwise comparisons are to be read as columns *vs.* rows: species are more frequent in the ecoregion (or group) heading a column than in the ecoregion of the intersecting row. Only species with 2ΔI>3.84 are shown and those with values of 2ΔI>6.63 are shown in bold.

**Figure 6:**
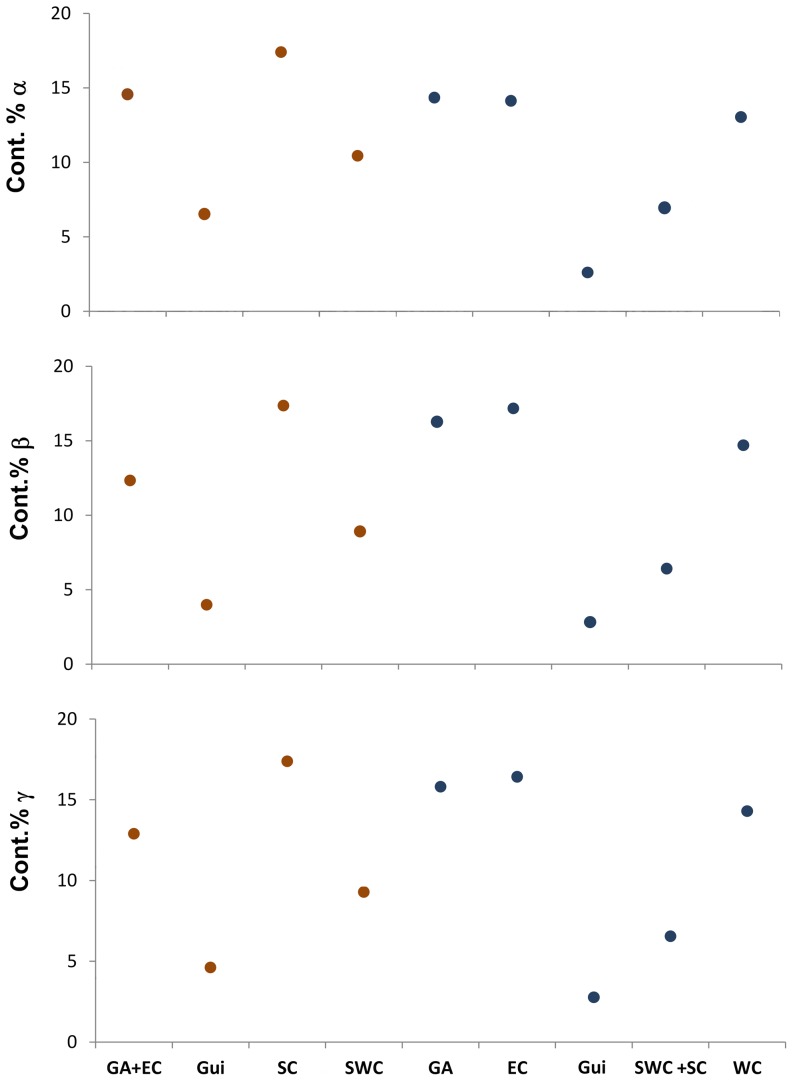
Relative contribution of regions to the diversity of deep-water corals for the upper continental shelf (red) and continental slope (blue) in the Caribbean Basin. EC: Eastern Caribbean, Gui: Guianian, SC: Southern Caribbean, SWC: Southwestern Caribbean, WC: Western Caribbean.

The differences in species composition among ecoregions suggest that the diversity of corals in the Caribbean Sea is a product of a high level of species turnover between ecoregions. This hypothesis is supported by the analysis of the additive partitioning of overall diversity (gamma diversity) into local diversity (alpha diversity) and turnover (beta diversity) [57]. Beta diversity was the most important component of gamma diversity (D_st_ = 0.752). Moreover, ecoregions varied in their contribution to gamma and beta diversity. For the upper continental shelf, the Southern Caribbean showed the greatest contribution to overall alpha, beta and gamma diversity, followed by the Greater Antilles-Eastern Caribbean group. On the continental slope, the Greater Antilles, Western Caribbean and Eastern Caribbean ecoregions had important contributions to all components of diversity, and together they represented 41.1% of the gamma diversity (Fig. 6). The contribution of the Guianian ecoregion and the Southwestern-Southern Caribbean group were lower than other ecoregions in this depth range.

## Discussion

Based on analyses of species accumulation curves, the present study confirms previous suggestions, based on species depth ranges, that coral diversity is higher on the continental slope in the Caribbean Sea [34]. Furthermore, the present study provided a robust test of these differences given the sampling coverage of the *Pillsbury* expeditions. An increase in faunal richness in the deep waters of the continental slope or rise relative to continental shelf habitats has been found by numerous studies [7], [32], [34], [60]–[65], although these differences depend on the basins, taxa and types of communities studied. The critical views and evidence furnished by Gray *et al*. [30] show exceptions to this trend and emphasize the effect of the area sampled on some estimates. For deep-water corals, niche-specific boundaries could contribute to the observed diversity pattern. According to Cairns [1], 63.4% of azooxanthellate Scleractinia live between 200–1000 m, 14.7% live between 1000–2000 m and 52.9% live between 50–200 m (these percentages sum to a value greater than 100% because the original values included species that inhabit more than one of the specified depth ranges).

Although the diversity of the continental rise and abyssal depths was not estimated due to insufficient sampling, few coral species were present below 2000 m (Fig. 2). In these depth ranges, data records indicate that the species present were antipatharians (*Antipathes umbratica*, *Abyssopathes lyra*, *Bathypathes seculata, Bathypathes* sp. and *Cirrhipathes* sp.), scleractinians (*Deltocyathus italicus, Fungiacyathus cripus, F. marenzelleri*) and one alcyonarian (*Thesea* sp.). Despite, Briggs *et al.* [33] did not report any deep-water coral species in samples from the Venezuelan Basin at depths between 3411 and 5062 m [33], *Pillsbury* data records indicated the presence of *Thesea* sp., *F. cripus* and *A. lyra* at about 4200, 4500 and 5400 m, respectively. According to Guinotte *et al.* [6] the aragonite saturation horizon (ASH) in the waters of the northwestern Atlantic is located between 2000–3000 m, limiting (although not prohibiting) the development of carbonate structures below this zone. Likewise, globally, only 4.7% of the deep-water scleractinians are found at depths below 2000 m [1] and global models suggest that the most suitable habitats for framework-forming corals and octocorals worldwide are the continental shelf and the continental slope [28], [29]. Based on the current data, a unimodal curve can be used to describe the diversity of deep-water corals, with high values of richness on the continental slope and lower values in shallower waters and at bathyal depths, which is consistent with the pattern proposed by Dawson [34] for azooxanthellate Scleractinia in the Caribbean and surrounding regions based on depth ranges of the species.

This study detected a turnover of coral species composition associated with large depth ranges, in addition to the effect of depth. Zonation by depth is a widespread pattern in the deep-water biota [66]. The overall diversity (gamma diversity) of deep-water corals depends on the number of species at each depth interval and the rate of species replacement between depth intervals. Changes in composition along depth ranges could be related to differences in habitat characteristics rather than depth between the upper continental shelf and the continental slope. Despite its depth, the continental slope shows high topographic and hydrographic heterogeneity [32], [67], variation in slope inclination and seafloor, habitats (ridges, seamounts and tablemounts [9], [20], [27]), as well as in tectonic, terrestrial and oceanic influences that shape physical configurations, which have different hydrographic conditions such as water mass, temperature and carbon flux. The Caribbean continental slope is bathed by at least four water masses at different depths and points of entrance in the basin [12], [13]. Furthermore, because both topographic and hydrographic conditions have an influence in the distribution of deep-water corals [28], [29], [67], [68], heterogeneity in these conditions could enhance fauna diversity [69], [70].

The differences detected between ecoregions in species composition on both the upper continental shelf and the continental slope support the hypothesis that the deep-water fauna of the Caribbean Basin and nearby waters reflect a heterogeneous species composition at a regional scale. This finding contrasts with previous work [34] that suggests the absence of differences in composition of deep-water corals between ecoregions in the Caribbean basin, for both depth ranges. Nevertheless, similarity between nearby ecoregions, forming larger groups (i.e., Greater Antilles-+Eastern Caribbean on the continental shelf and the Southwestern Caribbean-Southern Caribbean group), and between distant ecoregions (i.e., Greater Antilles and Southern ecoregions on the continental slope) suggests a wide connectivity at both depth ranges. Gene flow and connectivity of deep-water corals has been associated with water mass stratification [11], [12]. Henry [2] postulated that widespread circulation of the Antarctic Intermediate Water in the Caribbean could enhance larval flow of deep-water corals through the basin.

The ecoregions obtained by the present study differs from those proposed by Cairns & Chapman [23] for deep-water Scleractinia; however both studies show heterogeneity in the Caribbean Basin, and affinities between ecoregions. The discrepancies related to ecoregions between the previous works [23], [34] and the present study mainly involve detection of within-ecoregion variation (in contrast with Dawson [34]), and the separation of the northern regions, sorted previously [23] as “insular” cluster, which could be explained by the increase of taxa coverture (by inclusion of Alcyonaria and Antipatharia), data source (use of samples from one expedition survey) and statistical approach (analysis of variance instead of hierarchical clustering). Although a cluster analysis of checklist could be preferable by the selection of a “complete” list of species, using records from different surveys could introduce selection bias due to the area and ecoregion(s) covered in each survey and due to pooling data without estimating the variation between samples within the same unit (i.e., ecoregions). Moreover, data manipulation, such as excluding endemic species [34], could overlook an important component of diversity. Although endemism could be an artifact of sampling coverage and collection methods [71], excluding them may actually contribute to the homogeneity previously reported for the Caribbean basin [34].

Despite differences in sampling design or checklist studies, the analysis of indicator species (*I*-test, Table 4) was generally consistent with other studies. On the upper continental shelf, *Madracis myriabilis* has widespread distribution in the Caribbean but is more frequent in the Antillean arc [21], although there are exceptions [21], [22], [25]. *Nicella guadalupensis* have a similar pattern of distribution; there are few records of this species in offshore reefs from Colombia [24], [44] and Venezuela [72], compared to its distribution along the Antilles [72]. There are species that are frequent in the Southern Caribbean but not in the Southwestern Caribbean, such as *Aphanipathes pedata*, which has not been reported in Colombian Shelf waters to date [44]. Similarly, *Balanophyllia dineta* and *Bellonella rubistella* have been reported with a frequency lower than 5% for samples from Colombia [44], compared to 40–43% from the Southern Caribbean in the present study. The occurrence of *Cladocora debilis* in the Southern Caribbean in contrast with the Greater Antilles is consistent with the distribution pattern of this species [22], but differences in frequency between the Southern and Southwestern Caribbean have not been reflected in recent samples [25], [44]. Other taxa such as *Leptogorgia* sp. and *Thesea* sp. cannot be compared because they contain various species with different patterns of distribution. Regarding the frequent species in the Southwestern Caribbean, *Diodogorgia* sp., could be related with the only species of the genus reported, *Diodogorgia nodulifera*, which is a widespread species on the Colombian shelf [44].

Although the continental slope had higher diversity, only a few species showed contrasting frequencies, in part because the assemblages showed less variation between ecoregions at this depth (Table 3), and the variation could be enhanced by infrequent species. The frequency of *Stephanocyathus* species (*S. paliferus* and *S. diadema*) in the Southwestern Caribbean-Southern Caribbean group is supported by recent samples in Colombian deep waters [44], but both species show widespread distribution in the Caribbean [21]. However, *Fungicyathus symetricus* was found only in low frequencies in the Southern and Southwestern Caribbean ecoregions, being recorded only in the deep waters of offshore Colombian islands [44], compared to numerous records in the Eastern Caribbean [21].

The substantial contribution of beta diversity to overall diversity indicates that the high diversity of deep-water corals is a product of high turnover in assemblages between ecoregions and depth ranges. A potential explanation is that the observed turnover is associated with the interaction between environmental variation (topographic and hydrographic) and niche-specific conditions. Differences in deep-water fauna at global and regional scales have been related to environmental factors, including productivity, sediment heterogeneity, oxygen availability, hydrodynamics, slope and disturbance [28], [29], [32], [73]–[76]. In addition to the topographic variation, the Caribbean shows differences in POC flux [16], [18] and other sources of carbon and energy in the Caribbean can contribute directly to the benthos. These sources include hydrothermal vents, hydrocarbon seeps and mud volcanoes, all of which could affect faunal assemblages [77]–[79]. An approach to the hypothesis of environmental factors as causes of regional differences in coral composition requires correlative patterns of assemblages with environmental models, for both water mass characteristics and topographical attributes. Future work should attempt to determine if the patterns detected in corals are found in other megafaunal invertebrates (crustaceans, mollusks, echinoderms, etc.) from deep waters.

The importance of inter-regional beta diversity and the relative contributions of different ecoregions to gamma diversity highlight the need for joint efforts by different countries to facilitate the management and conservation of deep-water diversity. In general, the Caribbean Basin and nearby regions face potential direct anthropogenic impacts on the deep-water benthos produced by bottom trawl fisheries and oil exploitation [8], [80], [81]. Moreover, Guinotte *et al.* [6] predict that an increase in ocean acidification will reduce the ASH worldwide, decreasing the suitability of the ocean environment for deep-water corals. Efforts on regional and global scales are necessary to support the sustainable persistence of this fauna.

Recent studies have drawn attention to include deep-water environments in marine protected areas in the Caribbean [26], [44]. According to the present results, the assemblage of deep-water corals from the Caribbean are not uniform either in depth range or at the ecoregional scale, and the overall diversity depends largely on beta diversity. Therefore, an effective strategy for the conservation of diversity must include a broad initiative covering ecoregions and depth ranges. As consider the design of marine protected areas for deep waters in various ecoregions and define basin-scale standards for the exploitation of deep habitats. In addition, diversity may have patterns of variation at various scales within ecoregions, as shown by the spatial patterns in Colombia [24]. Intra-regional patterns, areal estimation [15], [82] and modeling of the environmental factors that ensure the sustainability of coral populations at regional scale are necessary for the proper understanding and management of this ecosystem.

## Supporting Information

File S1 Table S1. Records of deep-water corals recorded during the R/V *Pillsbury* expedition according to ecoregion and depth range.
(DOCX)

File S2 Sensitivity of Estimations to Taxonomic Resolution. Table S2. PERMANOVA for similarity in generic composition of deep-water corals (Sorensen's similarity index). Table S3. Type I error probabilities for pairwise *t*-tests of similarity in generic composition of deep-water corals between ecoregions. Fig.S1. Accumulative curve of genera (A) and the Chao2 estimator (B) for the upper continental shelf (red) and the continental slope (blue). Fig. S2. nm-MDS of similarity between stations based on Sorensen's similarity index (generic level), according to depth range.
(DOCX)

## References

[1] Cairns SD (2007) Deep-water corals: an overview with special reference to diversity and distribution of deep-water scleractinian corals. Bull Mar Sci 81(3): 311–322.

[2] Henry LA (2011) A deep-sea coral ‘gateway’ in the northwestern Caribbean. In: Palomares MLD, Pauly D Too Precious to Drill: the Marine Biodiversity of Belize, pp. 120–124. Fisheries Centre Research Reports 19(6). Fisheries Centre, University of British Columbia [ISSN 1198–6727].

[3] Mosher CV, Watling L (2009) Partners for life: a brittle star and its octocoral host. Mar Ecol Prog Ser 397: 81–88.

[4] Roberts JM, Wheeler AJ, Freiwald A (2006) Reefs of the deep: the biology and geology of cold-water coral ecosystems. Science 312: 543–547. 16645087 10.1126/science.1119861

[5] Clark MR, Tittensor D, Rogers AD, Brewin P, Schlacher T, et al. (2006) Seamounts, deep-sea corals and fisheries: vulnerability of deep-sea corals to fishing on seamounts beyond areas of national jurisdiction. UNEPWCMC, Cambridge, UK.

[6] Guinotte JM, Orr J, Cairns SD, Freiwald S, Morgan L, et al (2006) Will human induced changes in seawater chemistry alter the distribution of deep-sea scleractinian corals? Front Ecol Envirom 4: 141–146.

[7] Ramirez-Llodra E, Brandt A, Danovaro R, Escobar E, German CR, et al (2010) Deep, diverse and definitely different: unique attributes of the world's largest ecosystem. Biogeosci Disc 7: 2361–2485.

[8] White H, Hsing PY, Cho W, Shank T, Cordes EE, et al. (2012) Impact of the *deepwater horizon* oil spill on a deep-water coral community in the Gulf of Mexico. Proc Nat Acad Sci doi:10.1073/pnas.1118029109. PMC3528508 22454495

[9] Miloslavich P, Díaz JM, Klein E, Alvarado JJ, Díaz C, et al (2010) Marine biodiversity in the Caribbean: regional estimates and distribution patterns. PLoS ONE 5(8): e11916 10.1371/journal.pone.0011916 20689856 PMC2914069

[10] Spalding M, Fox H, Allen G, Davidson N, Ferdaña Z, et al (2007) Marine ecoregions of the world: a bioregionalization of coastal and shelf areas. BioScience 57: 573–583.

[11] Kitchingman A, Lai S (2005) Inferences on potential seamount locations from mid-resolution bathymetric data. In Morato T, Pauly D Seamounts: Biodiversity and Fisheries. 7–12.

[12] Gordon AL (1967) Circulation of the Caribbean Sea. J Geophys Res 72: 6207–6223.

[13] Schmitz WJ (1996) On the world ocean circulation. Vol 1. Wood Hole Oceanogr Inst Tech Rept WHOI-96-03.

[14] MacCready P, Johns WE, Rooth CG, Fratantoni DM, Watlington RA (1999) Overflow into the deep Caribbean: Effects of plume variability. J Geophys Res 104: 25913–25935.

[15] Hübscher C, Dullo C, Flögel S, Titschack J, Schönfeld J (2010) Contourite drift evolution and related coral growth in the eastern Gulf of Mexico and its gateways. Int J Earth Sci 99: S191–S206.

[16] Lutz MJ, Caldeira K, Dunbar RB, Behrenfeld MJ (2007) Seasonal rhythms of net primary production and particulate organic carbon flux to depth describe the efficiency of biological pump in the global ocean. J Geophys Res 112: , C10011. 10.1029/2006JC003706.

[17] Watlinga L, Guinotte J, Clark MR, Smith CR (2013) A proposed biogeography of the deep ocean floor. Prog Oceanog 111: 91–112.

[18] Müller-Karger FE, Castro RA (1994) Mesoscale processes affecting phytoplankton abundance in the southern Caribbean Sea. Cont Shelf Res 14: 199–221.

[19] Hellweger FL, Gordon AL (2002) Tracing Amazon river water into the Caribbean Sea. J Mar Res 60: 537–549.

[20] Lutz SJ, Ginsburg RN (2007) State of deep coral ecosystems in the Caribbean region: Puerto Rico and the U.S. Virgin Islands. In: Lumsden SE, Hourigan TF, Bruckner AW, Dorr G The State of Deep Coral Ecosystems of the United States. NOAA Tech Mem CRCP-3. Silver Spring MD 365 pp.

[21] Cairns SD (1979) The deep-water Scleractinia of the Caribbean and adjacent waters. Stud Fauna Curaçao & Caribb Is 57: 1–341.

[22] Cairns SD (2000) A revision of the shallow-water azooxanthellate Scleractinia of the Western Atlantic. Stud Nat Hist Caribb Reg 75: 1–231.

[23] Cairns SD, Chapman RE (2001) Biogeographic affinities of the North Atlantic deep-water Scleractinia. In: Willison JHM, Hall J, Gass SE, Kenchington ELR, Butler M, Doherty P Proceedings of the First International Symposium on Deep-Sea Corals, pp. 30–57. Ecology Action Centre, Halifax.

[24] Reyes J, Santodomingo N, Gracia A, Borrero-Pérez G, Navas G, et al. (2005) Southern Caribbean azooxanthellate coral communities off Colombia. Freiwald A, Roberts JM Cold-water Corals and Ecosystems. Springer-Verlag Berlin Heidelberg, pp 309–330.

[25] Santodomingo N, Reyes J, Gracia A, Martínez A, Ojeda G, et al. (2007) Azooxanthellate *Madracis* coral communities off San Bernardo and Rosario Islands (Colombian Caribbean). George RY, Cairns SD Conservation and adaptive management of seamount and deep-sea coral ecosystems. pp. 273–287. RSMAS, University of Miami.

[26] Urriago JD, Santodomingo N, Reyes J (2011) Formaciones coralinas de profundidad: criterios biológicos para la conformación de áreas marinas protegidas del margen continental (100–300 m) en el Caribe colombiano. Bol Invest Mar Cost 40 (1): 89–113.

[27] Tittensor DP, Baco A, Brewin P, Clark MR, Consalvey M, et al (2009) Predicting global habitat suitability for stony corals on seamounts. J Biogeogr 36: 1111–1128.

[28] Davies AJ, Guinotte JM (2011) Global Habitat Suitability for Framework-Forming Cold-Water Corals. PLoS ONE 6(4): e18483 10.1371/journal.pone.0018483 21525990 PMC3078123

[29] Yesson C, Taylor ML, Tittensor DP, Davies AJ, Guinotte JM, et al (2012) Global habitat suitability of cold-water octocorals. J Biogeogr 39: 1278–1292.

[30] Gray JS, Poore GCB, Ugland KI, Wilson RS, Olsgard F, et al (1997) Coastal and deep-sea benthic diversities compared. Mar Ecol Progr Ser 159: 97–103.

[31] Grassle JF, Maciolek NJ (1992) Deep-sea species richness: regional and local diversity estimates from quantitative bottom samples. Amer Nat 139: 313–341.

[32] Levin LA, Etter RJ, Rex MA, Gooday AJ, Smith CR, et al (2001) Environmental influences on regional deep-sea species diversity. Ann Rev Ecol Syst 32: 51–93.

[33] Briggs KB, Richardson MD, Young DK (1996) The classification and structure of megafaunal assemblages in the Venezuelan Basin, Caribbean Sea. J Mar Res 54: 705–730.

[34] Dawson JP (2002) Biogeography of azooxanthellate corals in the Caribbean and surrounding areas. Coral Reefs 21: 27–40.

[35] Voss GL (1966) Narrative of cruise P-6607 of the R/V John Elliot Pillsbury to the Southwestern Caribbean, July 7–22, 1966. Technical Report. RSMAS. University of Miami. 39 pp.

[36] Voss GL (1971a) Narrative of R/V John Elliot Pillsbury cruise P-7106 to the Nares Abyssal and Puerto Rico Trench, 26 June–13 July. Technical Report. RSMAS. University of Miami. 30 pp.

[37] Voss GL (1971b). Narrative of R/V John Elliot Pillsbury cruise P-7106 to Central America, 20 January–5 February 1971. Technical Report. RSMAS. University of Miami. 31 pp.

[38] Voss GL (1977) Study of the macrofauna of the tropical Western Atlantic. In Stewart HB Cooperative Investigations of the Caribbean and Adjacent Regions II. Symposium on Progress in Marine Research in the Caribbean and Adjacent Regions. Papers in Fisheries, Aquaculture and Marine Biology. FAO Fish Rep 200.

[39] Staiger J (1968a) Narrative of R/V John Elliot Pillsbury cruise P-6806 to the Southern Caribbean, July 6–August 3, 1968. Technical Report. RSMAS. University of Miami. 70 pp.

[40] Staiger J (1968b) Narrative of R/V John Elliot Pillsbury cruise P-6802 to Arrowsmith Bank and Northwestern Caribbean, March 11–March 26. Technical Report. RSMAS. University of Miami. 25 pp.

[41] Staiger J (1969) Narrative of R/V John Elliot Pillsbury cruise P-6907 to the Antillean Arc, June 27–July 31, 1969. Technical Report. RSMAS. University of Miami. 64 pp.

[42] Staiger J, Voss G (1970) Narrative of R/V John Elliot Pillsbury, cruise P-7006 to Hispañola and Jamaica, June 26 to July, 1970. Technical Report. RSMAS. University of Miami. 56 pp.

[43] McClain CR, Hardy SM (2010) The dynamics of biogeographic ranges in the deep sea. Proc R Soc B doi:10.1098/rspb.2010.1057. PMC2982252 20667884

[44] Santodomingo N, Reyes J, Flórez P, Chacón-Gómez IC, van Ofwegen LP, et al (2013) Diversity and distribution of azooxanthellate corals in the Colombian Caribbean. Mar Biodiv 43: 7–22.

[45] Chao A (1987) Estimating the population size for capture-recapture data with unequal chatchability. Biometrics 43: 783–791. 3427163

[46] Colwell RK (2013) EstimateS: Statistical estimation species richness and shared species from samples. Version 9. http://viceroy.eeb.uconn.edu/estimates/.

[47] Tittensor DP, Mora C, Jetz W, Lotze HK, Ricard D, et al. (2010) Global patterns and predictors of marine biodiversity across taxa. Nature 466: doi:10.1038/nature09329. 20668450

[48] Gray J (2000) The measurement of marine species diversity, with an application to the benthic fauna of the Norwegian continental shelf. J Exp Mar Biol Ecol 250: 23–49. 10969162 10.1016/s0022-0981(00)00178-7

[49] Paterson GLJ, Wilson GDF, Cosson N, Lamont PA (1998) Hessler and Jumars (1974) revisited: abyssal polychaete assemblages from the Atlantic and Pacific. Deep-Sea Res 45: 25–251.

[50] Ugland K, Gray JS, Ellingsen KE (2003) The species-accumulation curve and estimation of species richness. J Anim Ecol 72: 888–897.

[51] Chazdon RL, Colwell RK, Denslow JL,Guariguata ML (1998) Statistical methods for estimating species richness of woody regeneration in primary and secondary rain forest of Northeastern Costa Rica. In Dallmeier F, Comiskey JA p 285–309 Forest biodiversity research, monitoring and modeling. UNESCO, Paris.

[52] Colwell RK, Mao CX, Chang J (2004) Interpolating, extrapolating, and comparing incidence-based species accumulation curves. Ecology 85: 2717–2727.

[53] Guerra-Castro EJ (2012) Diversidad de especies, patrones y procesos estructurales de las comunidades incrustrantes asociadas a las raíces de mangle rojo *Rhizophora mangle* L. PhD Thesis IVIC, Venezuela. 309 pp.

[54] Anderson MJ (2001) A new method for non-parametric multivariate analysis of variance. Austral Ecol 26: 32–46.

[55] Anderson M, Gorley R, Clarke K (2008) PERMANOVA for PRIMER: Guide to software and statistical methods. Plymouth: PRIMER-E Ltd. 214.

[56] Field JG, Clarke KR, Warwick RM (1982) A practical strategy for analyzing multispecies distribution patterns. Mar Ecol Prog Ser 8: 37–52.

[57] Lu HP, Wagner HH, Chen XY (2007) A contribution diversity approach to evaluate species diversity. Basic Appl Ecol 8: 1–12.

[58] Cairns SD (1986) A revision of the northwest Atlantic Stylasteridae (Coelenterata: Hydrozoa). Smithson Contr Zool 418: 1–131.

[59] Cairns SD (2011) Global Diversity of the Stylasteridae (Cnidaria: Hydrozoa: Athecatae). PLoS ONE 6(7): e21670 10.1371/journal.pone.0021670 21799741 PMC3142106

[60] Sanders HL (1968) Marine benthic diversity: a comparative study. Amer Nat 102: 243–282.

[61] Dayton PK, Hessler RR (1972) Role of biological disturbance in maintaining diversity in the deep sea. Deep-Sea Res 19: 199–208.

[62] Rex MA (1973) Deep-sea species diversity: decrease gastropod diversity at abyssal depths. Science 181: 1051–1053. 17731267 10.1126/science.181.4104.1051

[63] Grassle JF (1991) Deep-sea benthic biodiversity. Bioscience 41: 464–469.

[64] Snelgrove PVR, Smith CR (2002) A riot of species in an environmental calm: The paradox of the species-rich deep sea. Oceanogr Mar Biol Annu Rev 40: 311–342.

[65] Stuart CT, Rex MA, Etter RJ (2003) Large-scale spatial and temporal patterns of deep-sea benthic species diversity. In Tyler PA Ecosystems of the Deep Oceans, Ecosystems of the World. Elsevier, Amsterdam. Pp. 295–313.

[66] Carney RS (2005) Zonation of deep biota on continental margins. Oceanogr Mar Biol Annu Rev 43: 211–278.

[67] Arantes RCM, Castro CB, Pires DO, Seoane JCS (2009) Depth and water mass zonation and species associations of cold-water octocoral and stony coral communities in the southwestern Atlantic. Mar Ecol Prog Ser 397: 71–79.

[68] Miller KJ, Rowden AA, Williams A, Häusserman V (2011) Out of their depth? Isolated deep populations of the cosmopolitan coral *Desmophyllum dianthus* may be highly vulnerable to environmental change. PLoS One 6: e19004. doi:10.1371/journal.pone.0019004. PMC3097177 21611159

[69] Levin LA, Dayton PK (2009) Ecological theory and continental margins: where shallow meets deep. Trends Ecol Evol, doi:10.1016/j.tree.2009.04.012. 19692143

[70] Priede IG, Godbold JA, King NJ, Collins MA, Bailey DB, et al (2010) Deep-sea demersal fish species richness in the Porcupine Seabight, NE Atlantic Ocean: global and regional patterns. Mar Ecol 31: 247–260.

[71] Clark MR, Schlacher TA, Rowden AA, Stocks KI, Consalvey M (2012) Science priorities for seamounts: research links to conservation and management. PLoSONE 7 (1): e29232. doi:10.1371/journal.pone.0029232. PMC3261142 22279531

[72] Cairns SD (2007) Studies on the western Atlantic Octocorallia (Gorgonacea: Ellisellidae). Part 7: The genera *Riisea* Duchassaing & Michelotti, 1860 and *Nicella* Gray, 1870. Proc Biol Soc Wash 120: 1–38.

[73] Leverette TL, Metaxas A (2005) Predicting habitat for two species of deep-water coral on the Canadian Atlantic continental shelf and slope. In Freiwald A, Roberts JM Cold-water corals and ecosystems. pp. 467–479. Springer-Verlag, Berlin.

[74] Bryan TL, Metaxas A (2006) Distribution of deep-water corals along the North American continental margins: relationships with environmental factors. Deep-Sea Res I 53: 1865–1879.

[75] Bryan TL, Metaxas A (2007) Predicting suitable habitat for deep-water gorgonian corals on the Atlantic and Pacific Continental Margins of North America. Mar Ecol Progr Ser 330: 113–126.

[76] Woodby D, Carlile D, Hulbert L (2009) Predictive modeling of coral distribution in the Central Aleutian Islands, USA. Mar Ecol Progr Ser 397: 227–240.

[77] Gracia A, Rangel-Buitrago N, Sellanes J (2012) Methane seep molluscs from the Sinu-San Jacinto fold belt in the Caribbean Sea of Colombia. J Mar Biol Assoc UK 92: 1367–1377.

[78] Connelly DP, Copley JT, Murton BJ, Stansfield K, Tyler PA, et al (2012) Hydrothermal vent fields and chemosynthetic biota on the world's deepest seafloor spreading centre. Nat Commun 3: 620 10.1038/ncomms1636 22233630 PMC3274706

[79] German CR, Ramirez-Llodra E, Baker MC, Tyler PA (2011) the ChEss Scientific Steering Committee (2011) Deep-Water Chemosynthetic Ecosystem Research during the Census of Marine Life Decade and Beyond: A Proposed Deep-Ocean Road Map. PLoS ONE 6(8): e23259 10.1371/journal.pone.0023259 21829722 PMC3150416

[80] García E, Cróquer A, Bastidas C, Bone D, Ramos R (2011) First environmental monitoring of offshore gas drilling discharges in the Deltana Platform, Venezuela. Cs Mar 37(2): 141–155.

[81] Jones DOB, Cruz-Motta JJ, Bone D, Kaariainen JI (2012) Effects of oil drilling activity on the deep water megabenthos of the Orinoco Fan, Venezuela. J Mar Biol Assoc UK 92: 245–253.

[82] Reed JK, Messing C, Walker BK, Brooke S, Correa TBS, et al (2013) Habitat characterization, distribution, and areal extent of deep-sea coral ecosystems off Florida, Southeastern U.S.A. Caribb J Sci 47: 13–30.

