# Supplementary material for: Patterns of Deep-Water Coral Diversity in the Caribbean Basin and Adjacent Southern Waters: An Approach based on Records from the R/V Pillsbury Expeditions
Source: PLoS One. 2014 Mar 26;9(3):e92834. doi: 10.1371/journal.pone.0092834 (PMC3966830; doi:10.1371/journal.pone.0092834)
Supplement: File S1 — Table S1. Records of deep-water corals recorded during the R/V Pillsbury expedition according to ecoregion and depth range. (DOCX) [file pone.0092834.s001.docx]

Supporting information 2

Sensitivity of Estimations to Taxonomic Resolution

Consistent with the comparison of species richness on the upper continental shelf and the continental slope, more taxa were predicted at the generic level for the continental slope compared to the upper continental shelf (Fig. 1). Despite overlapping 95% confidence interval limits, the cumulative genus curves according to the cumulative number of genera and the Chao2 estimator show differences between the two depth ranges (cumulative genera: t_999_= 73.2, p< 0.0001; Chao2 estimator: t_999_= 48.45, p< 0.0001).

There were differences in assemblage composition at the generic level and they were related to the interaction between depth range and ecoregion identity, in addition to effects associated with the depth of each station (PERMANOVA, Table 1). The MDS plot for the upper continental shelf and the continental slope shows a pattern similar to that seen for taxa identified to the species level (Fig. 2). RELATE analysis (Spearman) shows a high correlation between resemblance matrices at the specific and generic levels (*ρ*= 0.848, p= 0.001). The pairwise *t*-tests between regions (Table 2) show a pattern of differences similar to that detected at the species level, both for the upper continental shelf and continental slope, except for differences between the Guianian and Western Caribbean ecoregions on the continental slope that were not significant although close to the critical value (Table 2).

In conclusion, even in the extreme scenario that all identifications to species level were invalid (which is not possible due the indisputable expertise of taxonomists), the general patterns persisted. Therefore, separation of samples identified at the generic level into different species did not greatly affect the analysis, even if they all had belonged to a previously described species in the database.

Table S1. PERMANOVA for similarity in generic composition of deep-water corals (Sorensen’s similarity index). Significant differences are shown in bold.

| Source | df | SS | MS | Pseudo-F | P(perm) | Perms |
| --- | --- | --- | --- | --- | --- | --- |
| Depth (co) | 1 | 72634 | 72634 | 19.468 | **0.0001** | 9905 |
| Depth Range: De | 1 | 24104 | 24104 | 6.4607 | **0.0001** | 9880 |
| Ecoregion: Er | 5 | 41872 | 8374.3 | 2.2446 | **0.0001** | 9804 |
| De x Er | 5 | 35261 | 7052.2 | 1.8902 | **0.0001** | 9803 |
| Residuals | 175 | 652900 | 3730.8 |  |  |  |
| Total | 187 | 826770 |  |  |  |  |

Table S2. Type I error probabilities for pairwise *t*-tests of similarity in generic composition of deep-water corals between ecoregions. Significant differences are shown in bold. Samples with low sampling effort are shown in gray. *marginal p-value.

| Upper continental shelf | | |  |  |  |
| --- | --- | --- | --- | --- | --- |
|  | GA | EC | Gui | SC | SWC |
| EC | 0.1457 |  |  |  |  |
| Gui | **0.0014** | 0.0337 |  |  |  |
| SC | **0.0001** | **0.0007** | 0.1078 |  |  |
| SWC | **0.0001** | **0.0071** | 0.1384 | **0.0001** |  |
| WC | 0.3257 | 0.2298 | 0.2318 | 0.1615 | 0.2046 |
|  |  |  |  |  |  |
| Continental slope | |  |  |  |  |
|  | GA | EC | Gui | SC | SWC |
| EC | 0.2624 |  |  |  |  |
| Gui | 0.0715 | **0.0045** |  |  |  |
| SC | 0.2498 | **0.0057** | 0.4340 |  |  |
| SWC | 0.0099 | **0.0001** | 0.0796 | 0.1243 |  |
| WC | 0.0334 | 0.2971 | 0.0087* | **0.0005** | **0.0001** |


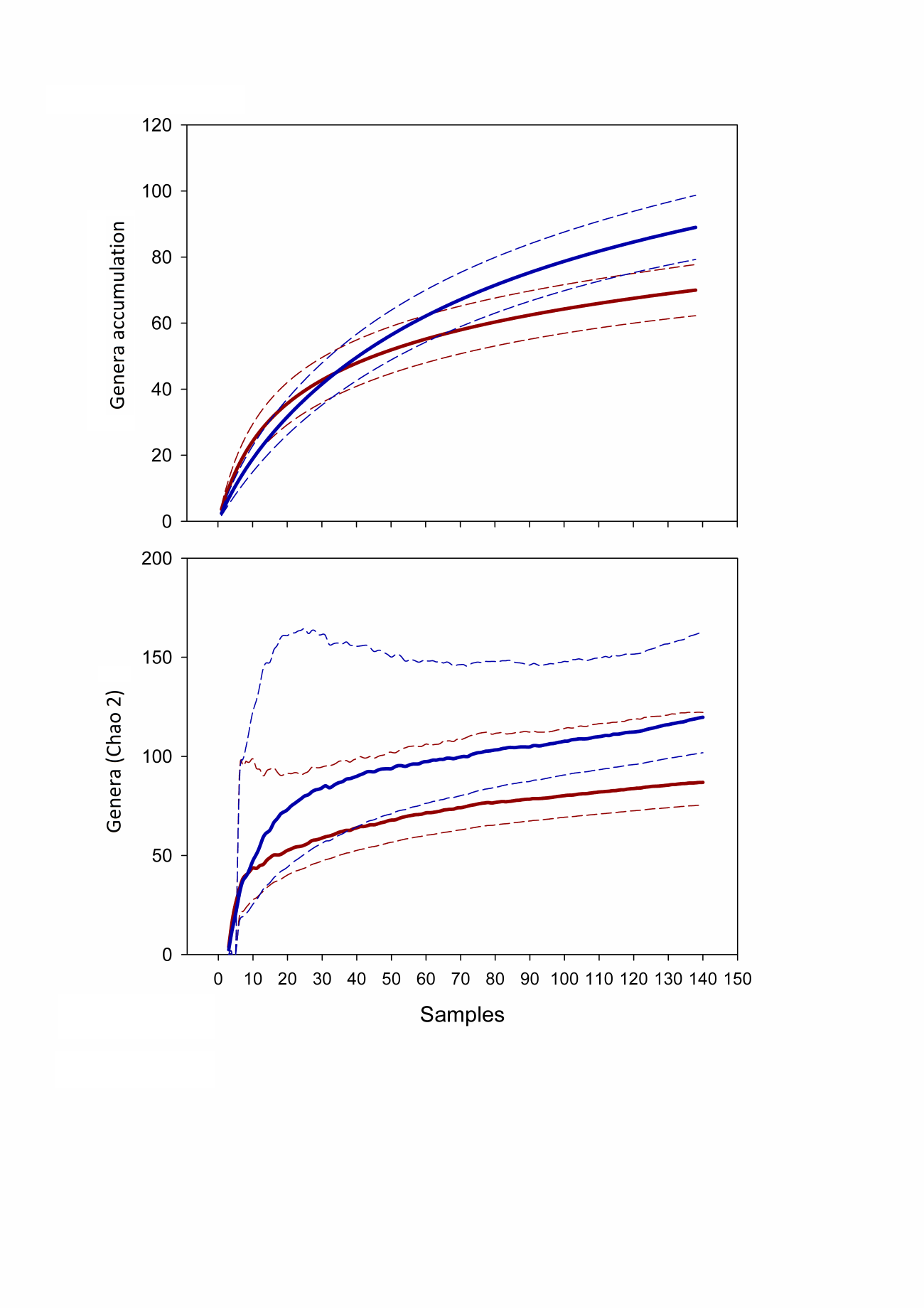


Fig.S1. Accumulative curve of genera (A) and the Chao2 estimator (B) for the upper continental shelf (red) and the continental slope (blue). Dashed lines represent 95% confidence intervals.


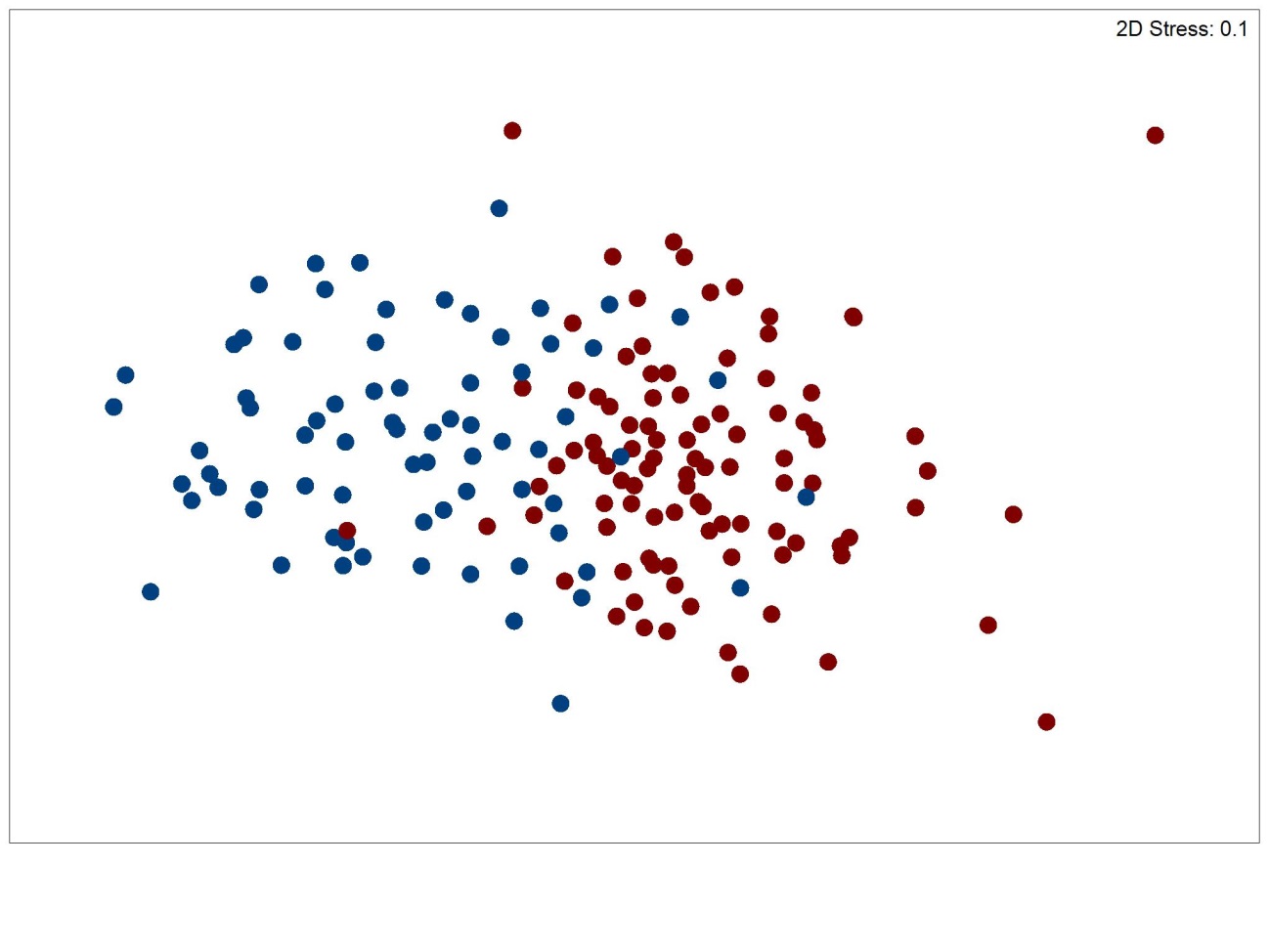


Fig. S2. nm-MDS of similarity between stations based on Sorensen’s similarity index (generic level), according to depth range. Red circles, upper continental shelf; blue circles, continental slope. Five outliers were excluded.
