# Supplementary material for: Patterns of Deep-Water Coral Diversity in the Caribbean Basin and Adjacent Southern Waters: An Approach based on Records from the R/V Pillsbury Expeditions
Source: PLoS One. 2014 Mar 26;9(3):e92834. doi: 10.1371/journal.pone.0092834 (PMC3966830; doi:10.1371/journal.pone.0092834)
Supplement: File S2 — Sensitivity of Estimations to Taxonomic Resolution. Table S2. PERMANOVA for similarity in generic composition of deep-water corals (Sorensen's similarity index). Table S3. Type I error probabilities for pairwise t-tests of similarity in generic composition of deep-water corals between ecoregions. Fig.S1. Accumulative curve of genera (A) and the Chao2 estimator (B) for the upper continental shelf (red) and the continental slope (blue). Fig. S2. nm-MDS of similarity between stations based on Sorensen's similarity index (generic level), according to depth range. (DOCX) [file pone.0092834.s002.docx]

Table S3. Records of deep-water corals recorded during the R/V *Pillsbury* expedition according to ecoregion and depth range. Ecoregions: GA: Greather Antilles; EC Eastern Caribbean; SC Southern Caribbean; SWC Southwestern Caribbean; WC Western Caribbean; Gui Guianian. Depth ranges: U, upper continental shelf (60-200 m); C, Continental Slope; B Bathyal depths (2000-4000 m), A, Abyssal depths (4000-6000 m).

|  |  |  | Region | GA | | | | EC | | SC | | | SWC | | WC | | Gui | |
| --- | --- | --- | --- | --- | --- | --- | --- | --- | --- | --- | --- | --- | --- | --- | --- | --- | --- | --- |
| Order | Family | Species | Depth Range | U | C | A | B | U | C | U | C | A | U | C | U | C | U | C |
| Alcyonacea | Acanthogorgiidae | *Acanthogorgia schrammi* (Duchassaing & Michelotti, 1864) | | 1 |  |  |  |  |  |  |  |  |  |  |  |  |  |  |
|  |  | *Acanthogorgia* sp. | | 1 | 2 |  |  |  | 6 | 7 | 2 |  | 1 |  |  | 1 | 2 |  |
|  | Alcyoniidae | *Anthomastus* sp. | |  |  |  |  |  |  |  | 1 |  |  |  |  |  |  | 2 |
|  |  | *Bellonella rubistella* Deichmann, 1936 | |  |  |  |  |  |  | 12 |  |  | 2 |  |  |  |  |  |
|  |  | *Bellonella* sp. | |  | 1 |  |  |  |  | 1 |  |  |  |  |  |  |  |  |
|  |  | *Stereonephthya portoricensis* (Hargitt, 1901) | |  |  |  |  | 1 |  | 2 |  |  |  |  |  |  | 1 |  |
|  | Anthothelidae | *Anthothela* sp. | |  | 1 |  |  |  | 1 |  |  |  |  |  |  |  |  |  |
|  |  | *Anthothela tropicalis* Bayer, 1961 | |  |  |  |  |  |  |  |  |  |  |  |  |  |  |  |
|  |  | *Diodogorgia* sp. | | 2 |  |  |  | 2 |  | 9 |  |  | 15 |  |  |  | 2 |  |
|  |  | *Iciligorgia schrammi* Duchassaing, 1870 | | 1 |  |  |  | 2 |  | 1 |  |  |  |  |  | 2 |  |  |
|  | Briareidae | *Briareum* sp. | | 1 |  |  |  |  |  |  |  |  |  |  |  |  |  |  |
|  | Chrysogorgiidae | *Chrysogorgia desbonni* Duchassaing & Michelotti, 1864 | |  |  |  |  |  |  |  |  |  |  |  |  | 2 |  |  |
|  |  | *Chrysogorgia elegans* (Verrill, 1883) | |  |  |  |  |  | 2 |  | 1 |  |  | 1 |  |  |  |  |
|  |  | *Chrysogorgia fewkesi* Verrill, 1883 | |  |  |  |  |  | 1 |  |  |  |  |  |  |  |  |  |
|  |  | *Chrysogorgia* sp. | |  |  |  |  |  |  |  |  |  |  |  |  |  |  | 1 |
|  |  | *Iridogorgia* sp. | |  | 1 |  |  |  |  |  |  |  |  |  |  |  |  |  |
|  |  | *Metallogorgia* sp. | |  |  |  |  |  |  |  |  |  |  |  |  |  |  |  |
|  |  | *Trichogorgia lyra* Bayer & Muzik, 1976 | |  |  |  |  |  |  |  |  |  | 3 |  |  |  |  |  |
|  |  | *Chrysopathes oligocrada* Opresko & Loiola, 2008 | |  |  |  |  |  |  |  |  |  |  |  |  | 1 |  |  |
|  |  | *Chrysopathes* sp. | |  |  |  |  |  |  |  | 1 |  | 1 |  |  | 2 |  | 1 |
|  | Clavulariidae | *Carijoa operculata* (Bayer, 1961) | |  |  |  |  |  |  | 1 |  |  |  |  |  |  |  |  |
|  |  | *Carijoa riisei* (Duchassaing & Michelotti, 1860) | |  |  |  |  |  |  | 3 |  |  | 2 |  |  |  |  |  |
|  |  | *Clavularia* sp. | |  |  |  |  |  |  | 1 |  |  |  |  |  |  |  |  |
|  |  | *Scleranthelia rugosa var. musiva* Studer, 1878 | | 2 | 1 |  |  |  |  | 1 |  |  |  |  |  |  | 1 |  |
|  |  | *Scleranthelia rugosa* (Pourtalès, 1867) | |  | 1 |  |  |  | 1 |  |  |  |  |  |  |  |  |  |
| Cont. |  |  | Region | GA | | | | EC | | SC | | | SWC | | WC | | Gui | |
| Order | Family | Species | Depth Range | U | C | A | B | U | C | U | C | A | U | C | U | C | U | C |
|  |  | *Telesto* sp. | | 1 |  |  |  | 1 |  | 7 |  |  | 2 |  | 1 | 1 |  |  |
|  |  | *Telestula* sp. | |  | 2 |  |  |  | 1 | 1 | 1 |  | 1 | 1 |  |  |  |  |
|  |  | *Telestula tubaria* Wright & Studer, 1889 | |  |  |  |  |  |  |  | 1 |  |  | 2 |  |  |  |  |
|  | Coralliidae | *Corallium* sp. | |  | 2 |  |  |  |  |  |  |  |  |  |  |  |  | 1 |
|  | Ellisellidae | *Ellisella schmitti* (Bayer, 1961) | | 1 |  |  |  |  |  |  |  |  | 2 |  |  |  |  |  |
|  |  | *Ellisella* sp. | |  |  |  |  | 6 |  | 13 |  |  | 6 |  |  | 1 | 6 |  |
|  |  | *Nicella americana* Toeplitz, 1919 | | 2 |  |  |  | 1 |  | 1 |  |  |  |  |  |  |  |  |
|  |  | *Nicella deichmannae* Cairns, 2007 | | 1 | 1 |  |  |  |  | 1 |  |  |  |  |  |  |  |  |
|  |  | *Nicella goreaui* Bayer, 1973 | |  |  |  |  | 1 |  | 2 |  |  | 6 |  |  |  |  |  |
|  |  | *Nicella gracilis* Cairns, 2007 | |  |  |  |  |  |  | 1 |  |  |  |  |  |  |  |  |
|  |  | *Nicella guadalupensis* (Duchassaing & Michelotti, 1860) | | 3 |  |  |  | 3 |  | 1 |  |  |  |  |  |  | 2 |  |
|  |  | *Nicella hebes* Cairns, 2007 | | 2 |  |  |  | 2 |  |  |  |  | 1 |  |  |  |  |  |
|  |  | *Nicella obesa* Deichmann, 1936 | |  | 2 |  |  |  | 2 |  | 1 |  |  |  |  |  |  |  |
|  |  | *Nicella robusta* Cairns, 2007 | | 1 |  |  |  |  |  | 1 | 1 |  | 1 |  |  | 1 |  |  |
|  |  | *Nicella* sp. | |  |  |  |  |  |  |  |  |  | 1 |  |  |  |  |  |
|  |  | *Nicella toeplitzae* Viada & Cairns, 2007 | | 1 |  |  |  |  |  | 1 |  |  | 1 |  |  |  |  |  |
|  |  | *Riisea paniculata* Duchassaing & Michelotti, 1860 | | 2 |  |  |  | 2 | 1 | 2 |  |  | 1 |  |  | 1 |  |  |
|  | Gorgoniidae | *Gorgonia* sp. | |  | 1 |  |  | 1 |  |  |  |  |  |  |  |  |  |  |
|  |  | *Gorgonia ventalina* Linnaeus, 1758 | |  | 1 |  |  |  |  |  |  |  |  |  |  |  |  |  |
|  |  | *Leptogorgia setacea* (Pallas, 1766) | |  |  |  |  |  |  | 2 |  |  |  |  |  |  |  |  |
|  |  | *Leptogorgia* sp. | |  |  |  |  |  |  | 7 |  |  | 2 |  |  |  | 2 |  |
|  |  | *Pseudopterogorgia* sp. | |  |  |  |  |  |  | 2 |  |  | 2 |  |  |  |  |  |
|  | Isididae | *Acanella eburnea* (Pourtalès, 1868) | |  |  |  |  |  | 1 |  |  |  |  |  |  |  |  |  |
|  |  | *Acanella* sp. | |  | 5 |  |  |  | 8 |  | 7 |  |  | 1 |  | 3 |  | 3 |
|  |  | *Caribisis simplex* Bayer & Stefani, 1987 | |  |  |  |  |  | 1 |  |  |  |  |  |  |  |  |  |
|  |  | *Keratoisis flexibilis* (Pourtales, 1868) | |  |  |  |  |  | 1 |  | 1 |  |  |  |  |  |  |  |
|  |  | *Keratoisis grayi* (Wright, 1869) | |  | 1 |  |  |  |  |  |  |  |  |  |  |  |  | 1 |
|  |  | *Keratoisis* sp. | |  |  |  |  |  | 1 |  |  |  |  |  |  |  |  |  |
|  |  | *Lepidisis caryophyllia* Verrill, 1883 | |  |  |  |  |  |  |  |  |  |  |  |  |  |  |  |
|  |  | *Lepidisis longiflora* Verrill, 1883 | |  |  |  |  |  | 1 |  |  |  |  |  |  |  |  |  |
|  |  | *Lepidisis simplex* (Verrill, 1883) | |  |  |  |  |  | 1 |  |  |  |  |  |  |  |  |  |
| Cont. |  |  | **Region** | GA | | | | EC | | SC | | | SWC | | WC | | Gui | |
| Order | Family | Species | Depth Range | U | C | A | B | U | C | U | C | A | U | C | U | C | U | C |
|  | Keroeididae | *Thelogorgia longiflora* Bayer, 1992 | |  |  |  |  |  |  |  |  |  | 1 |  | 1 |  |  |  |
|  |  | *Thelogorgia* sp. | | 2 |  |  |  |  |  |  |  |  |  |  |  |  |  |  |
|  |  | *Thelogorgia stellata* Bayer, 1992 | | 2 |  |  |  | 1 |  | 2 |  |  |  |  |  |  |  |  |
|  |  | *Thelogorgia studeri* Bayer, 1991 | |  | 1 |  |  |  |  |  |  |  |  |  |  |  |  |  |
|  |  | *Thelogorgia vossi* Bayer, 1991 | | 1 | 1 |  |  | 1 | 1 | 4 |  |  |  |  |  |  |  |  |
|  | Nephtheidae | *Neospongodes* sp. | | 1 |  |  |  | 4 |  | 1 |  |  |  |  |  | 1 |  |  |
|  |  | *Pseudodrifa nigra* (Pourtalès, 1868) | |  |  |  |  |  |  |  |  |  |  |  |  |  |  |  |
|  | Nidaliidae | *Nidalia dissidens* Verseveldt & Bayer, 1988 | |  |  |  |  |  | 1 |  |  |  |  |  |  |  |  |  |
|  |  | *Nidalia occidentalis* Gray, 1835 | | 1 | 1 |  |  |  |  | 5 |  |  | 3 |  |  | 1 | 1 |  |
|  |  | *Nidalia rubripunctata* Verseveldt & Bayer, 1988 | |  |  |  |  |  |  | 2 |  |  |  |  |  |  |  |  |
|  |  | *Nidalia* sp. | | 1 |  |  |  | 1 |  | 1 |  |  |  |  |  |  |  |  |
|  |  | *Siphonogorgia* sp. | | 3 | 3 |  |  |  | 2 | 4 |  |  | 2 |  |  |  |  |  |
|  | Plexauridae | *Acanthacis* sp. | |  |  |  |  | 1 |  |  |  |  |  |  |  |  |  |  |
|  |  | *Bebryce cinerea* Deichmann, 1936 | |  |  |  |  |  |  | 1 |  |  | 2 |  |  | 1 |  |  |
|  |  | *Bebryce grandis* Deichmann | |  |  |  |  |  |  |  |  |  | 1 |  |  | 1 |  |  |
|  |  | *Bebryce* sp. | | 2 | 2 |  |  | 3 |  | 4 | 1 |  | 2 |  |  |  | 1 |  |
|  |  | *Echinomuricea* sp. | |  |  |  |  |  |  | 1 |  |  | 1 |  |  | 2 |  |  |
|  |  | *Eunicea* sp. | |  | 1 |  |  |  |  |  |  |  |  |  |  |  |  |  |
|  |  | *Heterogorgia uatumani* Barreira e Castro, 1990 | |  |  |  |  |  |  | 1 |  |  |  |  |  |  |  |  |
|  |  | *Hypnogorgia pendula* Duchassaing & Michelotti, 1864 | |  |  |  |  | 1 |  |  |  |  |  |  |  |  |  |  |
|  |  | *Muricea laxa* Verrill, 1864 | |  | 1 |  |  |  |  |  |  |  |  |  |  |  |  |  |
|  |  | *Muricea pinnata* Bayer, 1961 | |  | 1 |  |  |  |  |  |  |  |  |  |  |  |  |  |
|  |  | *Muricea* sp. | |  | 1 |  |  |  |  | 2 |  |  |  |  |  |  | 1 |  |
|  |  | *Muriceides* sp. | |  |  |  |  |  | 1 | 1 |  |  |  |  |  |  | 1 |  |
|  |  | *Paramuricea* sp. | |  |  |  |  |  | 1 |  |  |  |  |  |  |  | 1 | 1 |
|  |  | *Placogorgia mirabilis* Deichmann, 1936 | |  |  |  |  |  |  |  |  |  |  |  |  | 1 |  |  |
|  |  | *Placogorgia* sp. | | 4 | 4 |  |  | 1 |  | 6 |  |  |  |  |  | 2 |  |  |
|  |  | *Plexaura flexuosa* Lamouroux, 1821 | |  | 1 |  |  |  |  |  |  |  |  |  |  |  |  |  |
|  |  | *Plexaura* sp. | |  | 1 |  |  |  |  |  |  |  |  |  |  |  |  |  |
|  |  | *Plexaurella* sp. | |  | 1 |  |  |  |  |  |  |  |  |  |  |  |  |  |
|  |  | *Scleracis guadalupensis* (Duchassaing & Michelotti, 1860) | |  |  |  |  |  |  |  |  |  |  |  |  | 1 |  |  |
| Cont. |  |  | Region | GA | | | | EC | | SC | | | SWC | | WC | | Gui | |
| Order | Family | Species | Depth Range | U | C | A | B | U | C | U | C | A | U | C | U | C | U | C |
|  |  | *Scleracis* sp. | | 1 |  |  |  | 2 |  | 4 |  |  | 7 |  |  | 2 | 1 |  |
|  |  | *Swiftia exserta* (Ellis & Solander, 1786) | |  |  |  |  |  |  | 4 |  |  | 4 |  |  |  | 3 |  |
|  |  | *Swiftia* sp. | | 1 | 1 |  |  |  |  | 1 | 1 |  |  |  |  |  |  |  |
|  |  | *Thesea* cf. *plana* Deichmann, 1936 | |  |  |  |  |  |  | 1 |  |  |  |  |  |  |  |  |
|  |  | *Thesea nivea* Deichmann, 1936 | |  |  |  |  |  | 1 |  |  |  |  |  |  |  |  |  |
|  |  | *Thesea nutans* Duchassaing & Michelotti, 1864 | |  |  |  |  | 1 |  | 1 |  |  | 2 |  |  | 1 |  |  |
|  |  | *Thesea* sp. | | 3 | 2 |  |  | 4 | 2 | 13 |  | 1 | 1 |  |  | 4 | 2 |  |
|  |  | *Villogorgia* sp. | | 1 | 2 |  |  | 4 | 1 | 2 |  |  | 5 |  |  | 1 | 1 |  |
|  | Primnoidae | *Acanthoprimnoa goesi* (Aurivillius, 1931) | |  |  |  |  |  |  |  |  |  |  |  | 2 | 1 |  |  |
|  |  | *Acanthoprimnoa pectinata* Cairns & Bayer, 2004 | |  |  |  |  |  |  |  |  |  |  |  |  | 2 |  |  |
|  |  | *Callogorgia* sp. | |  | 1 |  |  |  |  |  |  |  |  |  |  |  |  |  |
|  |  | *Candidella imbricata* (Johnson, 1862) | |  | 1 |  |  |  | 2 |  |  |  |  |  |  |  |  |  |
|  |  | *Narella bellissima* (Kukenthal, 1915) | |  |  |  |  |  | 1 |  |  |  |  |  |  |  |  |  |
|  |  | *Narella pauciflora* Deichmann, 1936 | |  | 1 |  |  |  | 1 |  |  |  |  |  |  |  |  |  |
|  |  | *Primnoella polita* Deichmann, 1936 | |  |  |  |  |  | 1 |  | 2 |  |  |  |  |  |  |  |
|  |  | *Thouarella bipinnata* Cairns, 2006 | |  |  |  |  |  |  |  |  |  |  |  |  |  |  | 1 |
| Antipatharia | Antipathidae | *Antipathes atlantica/affinis* complex | |  |  |  |  |  |  | 1 |  |  |  |  |  |  | 1 |  |
|  |  | *Antipathes barbadensis* Brook, 1889 | | 1 |  |  |  |  |  | 1 |  |  |  |  |  |  |  |  |
|  |  | *Antipathes caribbeana* Opresko, 1996 | |  |  |  |  |  |  |  |  |  |  | 1 |  |  |  |  |
|  |  | *Antipathes furcata* Gray, 1857 | | 3 |  |  |  | 1 | 1 |  |  |  |  |  |  |  | 2 |  |
|  |  | *Antipathes lenta* Pourtalès, 1871 | | 3 | 1 |  |  | 1 |  | 11 | 1 |  | 6 |  | 1 | 1 | 2 |  |
|  |  | *Antipathes pricea* | |  |  |  |  | 2 |  |  |  |  |  |  |  |  |  |  |
|  |  | *Antipathes rhipidion* Pax, 1916 | |  |  |  |  |  |  |  |  |  | 1 |  |  |  |  |  |
|  |  | *Antipathes tristis* (Duchassaing, 1870) | |  |  |  |  | 3 | 1 |  |  |  |  |  |  |  |  |  |
|  |  | *Antipathes umbratica* Opresko, 1996 | |  |  |  | 1 |  |  |  |  |  |  |  |  |  |  |  |
|  |  | *Cirrhipathes* sp. | | 2 |  |  | 1 | 1 |  | 5 |  |  | 1 |  |  |  |  | 1 |
|  | Aphanipathidae | *Acanthopathes humilis* (Pourtalès, 1867) | |  |  |  |  | 2 |  |  |  |  |  |  |  | 1 |  |  |
|  |  | *Acanthopathes thyoides* (Pourtalès, 1880) | |  |  |  |  | 1 |  | 1 |  |  |  |  |  |  |  |  |
|  |  | *Aphanipathes abietina* (Pourtalès, 1874) | | 1 | 1 |  |  | 3 | 2 | 1 |  |  |  |  |  | 1 |  |  |
|  |  | *Aphanipathes pedata* (Gray, 1857) | |  |  |  |  |  |  | 7 |  |  |  |  |  | 1 |  |  |
|  |  | *Aphanipathes salix* (Pourtalès, 1880) | |  |  |  |  | 2 |  |  |  |  | 1 |  |  |  |  |  |
| Cont. |  |  | Region | GA | | | | EC | | SC | | | SWC | | WC | | Gui | |
| Order | Family | Species | Depth Range | U | C | A | B | U | C | U | C | A | U | C | U | C | U | C |
|  |  | *Distichopathes disticha* Opresko, 2004 | |  |  |  |  |  |  |  |  |  |  |  |  | 1 |  |  |
|  |  | *Elatopathes abietina* (Pourtalès, 1874) | |  |  |  |  |  |  |  |  |  |  |  |  | 1 |  |  |
|  | Cladopathidae | Cladopathidae no id. | |  |  |  |  | 1 |  |  |  |  |  |  |  |  |  |  |
|  | Myriopathidae | *Plumapathes pennacea* (Pallas, 1766) | |  |  |  |  | 1 | 1 |  |  |  |  |  |  |  |  |  |
|  |  | *Tanacetipathes barbadensis* (Brook, 1889) | | 1 |  |  |  | 2 |  | 5 |  |  | 1 |  |  |  | 1 |  |
|  |  | *Tanacetipathes hirta* (Gray, 1857) | |  | 1 |  |  |  |  | 1 |  |  |  |  |  |  | 1 |  |
|  |  | *Tanacetipathes tanacetum* (Pourtalès, 1880) | |  |  |  |  | 3 |  | 2 |  |  | 1 | 1 |  | 1 | 1 |  |
|  |  | *Tanacetipathes thamnea* (Warner, 1981) | |  |  |  |  |  |  |  |  |  |  |  |  |  | 1 |  |
|  | Schizopathidae | *Abyssopathes lyra* (Brook, 1889) | |  |  | 1 |  |  |  |  |  |  |  |  |  |  |  |  |
|  |  | *Bathypathes alternata* Brook, 1889 | |  |  |  |  |  |  |  |  |  |  |  |  | 1 |  |  |
|  |  | *Bathypathes patula* Brook, 1889 | |  |  |  |  |  | 1 |  |  |  |  |  |  |  |  |  |
|  |  | *Bathypathes seculata* Opresko, 2005 | |  |  |  | 1 |  |  |  |  |  |  |  |  |  |  |  |
|  |  | *Bathypathes* sp. | |  |  | 1 | 1 |  |  |  |  |  |  |  |  |  |  |  |
|  |  | *Parantipathes larix* (Esper, 1788) | |  |  |  |  |  | 1 |  |  |  |  |  |  |  |  |  |
|  |  | *Parantipathes* sp. | |  | 1 |  |  |  | 2 |  |  |  |  |  |  | 2 |  |  |
|  |  | *Parantipathes tetrasticha* (Pourtalès, 1868) | |  | 1 |  |  |  |  |  |  |  |  |  |  |  |  |  |
|  |  | *Schizopathes affinis* Brook, 1889 | |  |  |  |  |  |  | 1 |  |  |  |  |  | 1 |  |  |
|  |  | *Stichopathes* sp1. | |  |  |  |  |  |  | 2 |  |  |  |  |  |  |  |  |
|  |  | *Stichopathes* sp2. | |  | 1 |  |  | 2 |  | 1 |  |  |  |  |  |  |  |  |
|  |  | *Taxipathes recta* Brook, 1889 | |  |  |  |  |  |  |  |  |  |  | 1 |  |  |  |  |
|  | Stylopathidae | *Stylopathes adinocrada* Opresko, 2006 | |  |  |  |  |  | 1 |  |  |  |  |  |  |  |  |  |
|  |  | *Stylopathes americana* (Duchassaing & Michelotti, 1860) | | 2 | 1 |  |  |  | 1 |  |  |  |  |  |  |  |  |  |
|  |  | *Stylopathes columnaris* (Duchassaing, 1870) | |  |  |  |  | 1 | 1 | 1 |  |  |  |  |  | 1 |  |  |
| Scleractinia | Caryophylliidae | *Coenocyathus parvulus* (Cairns, 1979) | |  | 1 |  |  |  |  |  |  |  |  |  |  |  |  |  |
|  |  | *Coenosmilia arbuscula* Pourtalès, 1874 | | 2 | 1 |  |  | 2 |  |  |  |  |  |  |  |  |  |  |
|  |  | *Crispatotrochus* cf. *cornu* (Moseley, 1881) | |  |  |  |  |  |  |  |  |  |  |  |  | 1 |  |  |
|  |  | *Dasmosmilia lymani* (Pourtalès, 1871) | |  |  |  |  |  |  | 1 |  |  |  |  |  |  |  |  |
|  |  | *Dasmosmilia prolifera* | |  |  |  |  |  |  | 1 |  |  |  |  |  |  |  |  |
|  |  | *Deltocyathus calcar* Pourtalès, 1874 | | 1 | 1 |  |  | 2 | 4 |  |  |  | 1 | 1 |  |  |  |  |
|  |  | *Deltocyathus eccentricus* Cairns, 1979 | |  | 2 |  |  |  | 4 |  | 1 |  |  | 2 |  | 2 |  |  |
|  |  | *Deltocyathus italicus* (Michelotti, 1838) | |  | 5 |  | 1 |  | 3 |  | 2 |  |  |  |  |  |  |  |
| Cont. |  |  | Region | GA | | | | EC | | SC | | | SWC | | WC | | Gui | |
| Order | Family | Species | Depth Range | U | C | A | B | U | C | U | C | A | U | C | U | C | U | C |
|  |  | *Deltocyathus moseleyi* Cairns, 1979 | |  |  |  |  | 1 | 2 |  | 1 |  |  |  |  |  |  |  |
|  |  | *Lophelia pertusa* (Linnaeus, 1758) | |  |  |  |  |  |  |  | 1 |  |  |  |  |  |  |  |
|  |  | *Oxysmilia rotundifolia* (Milne Edwards & Haime, 1848) | |  |  |  |  |  |  | 1 |  |  |  |  |  |  |  |  |
|  |  | *Paracyathus pulchellus* (Philippi, 1842) | |  | 1 |  |  |  |  | 2 |  |  |  |  |  |  |  |  |
|  |  | *Phacelocyathus flos* (Pourtalès, 1878) | | 1 |  |  |  |  |  |  |  |  | 1 |  |  |  |  |  |
|  |  | *Polycyathus* sp. | |  |  |  |  |  |  | 1 |  |  |  |  |  |  | 1 |  |
|  |  | *Rhizosmilia maculata* (Pourtalès, 1874) | |  | 1 |  |  | 3 | 1 | 2 |  |  | 1 |  |  | 1 |  |  |
|  |  | *Solenosmilia variabilis* Duncan, 1873 | |  | 1 |  |  |  | 2 |  |  |  |  |  |  |  |  |  |
|  |  | *Stephanocyathus (Odontocyathus) coronatus* (Pourtalès, 1867) | |  |  |  |  |  | 2 |  | 3 |  |  |  |  | 1 |  |  |
|  |  | *Stephanocyathus (Stephanocyathus) diadema* (Moseley, 1876) | |  | 6 |  |  |  |  |  | 5 |  |  | 6 |  |  |  | 2 |
|  |  | *Stephanocyathus (Stephanocyathus) laevifundus* Cairns, 1977 | |  |  |  |  |  | 1 |  |  |  |  | 1 |  |  |  |  |
|  |  | *Stephanocyathus (Stephanocyathus) paliferus* Cairns, 1977 | |  |  |  |  |  | 1 |  | 1 |  |  | 5 |  | 1 |  |  |
|  |  | *Tethocyathus variabilis*Cairns, 1979 | |  |  |  |  |  | 2 |  |  |  |  |  |  | 1 |  |  |
|  |  | *Thalamophyllia riisei* (Duchassaing & Michelotti, 1864) | |  | 1 |  |  |  |  |  |  |  | 1 |  |  | 1 |  |  |
|  |  | *Trochocyathus (Trochocyathus) fossulus* Cairns, 1979 | |  | 1 |  |  |  |  |  |  |  |  |  |  |  |  |  |
|  |  | *Trochocyathus (Trochocyathus) rawsonii* Pourtalès, 1874 | |  |  |  |  |  | 3 | 3 |  |  |  |  |  |  |  |  |
|  |  | *Trochocyathus* sp. | |  |  |  |  |  | 1 |  |  |  |  |  |  |  |  |  |
|  | Dendrophylliidae | *Balanophyllia (Balanophyllia) bayeri* Cairns, 1979 | |  |  |  |  |  |  |  |  |  |  |  |  | 1 |  |  |
|  |  | *Balanophyllia (Balanophyllia) cyathoides* (Pourtalès, 1871) | |  |  |  |  |  | 1 |  |  |  |  |  |  |  |  |  |
|  |  | *Balanophyllia (Balanophyllia) dineta* Cairns, 1977 | |  |  |  |  |  |  | 9 |  |  |  |  |  |  | 1 |  |
|  |  | *Balanophyllia (Balanophyllia) palifera* Pourtalès, 1878 | |  |  |  |  |  |  |  |  |  |  |  |  | 2 |  |  |
|  |  | *Balanophyllia (Balanophyllia) wellsi* Cairns, 1977 | |  |  |  |  |  |  | 1 |  |  |  |  |  |  |  |  |
|  |  | *Balanophyllia (Eupsammia) caribbeana* Cairns, 1977 | |  |  |  |  |  |  | 5 |  |  | 1 |  |  |  |  |  |
|  |  | *Balanophyllia (Eupsammia) pittieri* Vaughan, 1919 | |  |  |  |  |  |  |  |  |  | 3 |  |  |  |  |  |
|  |  | *Balanophyllia* sp. | |  |  |  |  |  |  |  |  |  | 1 |  |  | 2 | 1 |  |
|  |  | *Bathypsammia fallosocialis* Squires, 1959 | |  |  |  |  |  | 1 |  |  |  |  |  |  |  |  |  |
|  |  | *Cladopsammia manuelensis* (Chevalier, 1966) | |  |  |  |  |  |  |  |  |  |  |  |  | 1 |  |  |
|  |  | *Dendrophyllia alternata* Pourtalès, 1880 | |  |  |  |  |  | 1 |  |  |  |  |  |  |  |  |  |
|  |  | *Dendrophyllia* sp. | |  |  |  |  |  |  |  |  |  |  |  |  | 1 |  |  |
|  |  | *Eguchipsammia cornucopia* (Pourtalès, 1871) | |  | 1 |  |  |  | 2 |  |  |  |  |  |  |  |  |  |
|  |  | *Eguchipsammia gaditana* (Duncan, 1873) | |  |  |  |  |  |  |  |  |  |  |  |  | 1 |  |  |
| Cont. |  |  | Region | GA | | | | EC | | SC | | | SWC | | WC | | Gui | |
| Order | Family | Species | Depth Range | U | C | A | B | U | C | U | C | A | U | C | U | C | U | C |
|  |  | *Eguchipsammia strigosa* Cairns, 2000 | |  |  |  |  |  |  | 3 |  |  |  |  |  |  |  |  |
|  |  | *Enallopsammia rostrata* (Pourtalès, 1878) | |  | 3 |  |  |  | 1 |  |  |  |  |  |  |  |  |  |
|  |  | *Rhizosmilia gerdae* Cairns, 1978 | |  |  |  |  |  |  |  |  |  |  |  |  | 1 |  |  |
|  | Faviidae | *Cladocora debilis* Milne Edwards & Haime, 1849 | |  |  |  |  |  |  | 8 |  |  |  |  |  |  |  |  |
|  |  | *Manicina areolata* (Linnaeus, 1758) | |  |  |  |  |  |  | 2 |  |  |  |  |  |  | 1 |  |
|  |  | *Manicina* sp. | | 2 |  |  |  |  |  |  | 1 |  |  |  |  |  |  |  |
|  |  | *Montastraea cavernosa* Linnaeus, 1767 | | 1 |  |  |  |  |  |  |  |  |  |  |  |  |  |  |
|  |  | *Montastraea* sp. | |  | 2 |  |  |  |  |  |  |  |  |  |  |  |  |  |
|  | Flabellidae | *Flabellum (Ulocyathus) macandrewi* Gray, 1849 | |  |  |  |  |  |  |  | 1 |  |  |  |  |  |  |  |
|  |  | *Flabellum (Ulocyathus) moseleyi* Pourtalès, 1880 | |  | 1 |  |  |  | 3 |  | 2 |  |  | 1 |  |  |  |  |
|  |  | *Javania cailleti* (Duchassaing & Michelotti, 1864) | |  | 1 |  |  | 1 | 4 | 4 |  |  |  |  |  | 3 |  |  |
|  |  | *Polymyces fragilis* (Pourtalès, 1868) | |  |  |  |  |  | 2 |  |  |  |  |  |  | 1 |  |  |
|  | Fungiacyathidae | *Fungiacyathus (Bathyactis) crispus* (Pourtalès, 1871) | |  |  | 1 |  |  |  |  |  |  |  |  |  |  |  |  |
|  |  | *Fungiacyathus (Bathyactis) marenzelleri* (Vaughan, 1906) | |  |  |  | 3 |  |  |  |  |  |  |  |  |  |  |  |
|  |  | *Fungiacyathus (Bathyactis) symmetricus* (Pourtalès, 1871) | |  | 1 |  |  |  | 9 |  |  |  |  |  |  | 3 |  |  |
|  |  | *Fungiacyathus (Fungiacyathus) pusillus* (Pourtalès, 1868) | |  |  |  |  |  | 1 |  |  |  |  |  |  | 3 |  |  |
|  | Guyniidae | *Guynia annulata* Duncan, 1872 | | 1 |  |  |  |  |  |  |  |  |  | 1 |  |  |  |  |
|  | Mussidae | *Isophyllastraea rigida* (Dana, 1846) | |  | 1 |  |  |  |  |  |  |  |  |  |  |  |  |  |
|  |  | *Isophyllia* sp. | |  |  |  |  |  |  |  |  |  | 1 |  |  |  |  |  |
|  | Oculinidae | *Madrepora carolina* (Pourtalès, 1871) | |  |  |  |  |  |  |  |  |  |  |  |  |  |  |  |
|  |  | *Madrepora oculata* Linnaeus, 1758 | |  | 1 |  |  |  |  | 1 | 3 |  |  | 2 |  | 1 |  | 3 |
|  |  | *Oculina diffusa* Lamarck, 1816 | |  |  |  |  |  |  | 2 |  |  |  |  |  |  |  |  |
|  |  | *Oculina* sp. | |  |  |  |  |  |  |  |  |  | 1 |  |  |  |  |  |
|  | Pocilloporidae | *Madracis asperula* Milne Edwards & Haime, 1849 | | 1 |  |  |  |  |  | 2 |  |  |  |  |  |  |  |  |
|  |  | *Madracis decactis* (Lyman, 1859) | | 2 |  |  |  | 2 |  |  |  |  | 1 |  |  | 1 |  |  |
|  |  | *Madracis formosa* Wells, 1973 | |  | 1 |  |  |  |  |  |  |  |  |  |  |  |  |  |
|  |  | *Madracis myriaster* (Milne Edwards & Haime, 1849) | | 6 | 3 |  |  | 4 |  | 1 |  |  |  |  |  |  |  |  |
|  |  | *Madracis pharensis* (Heller, 1868) | |  |  |  |  |  |  | 1 |  |  | 1 |  |  |  |  |  |
|  |  | *Madracis* sp. | |  |  |  |  | 1 |  |  |  |  |  |  |  |  |  |  |
|  | Rhizangiidae | *Astrangia poculata* (Ellis & Solander, 1786) | |  |  |  |  |  |  |  |  |  |  |  |  |  | 1 |  |
|  |  | *Astrangia solitaria* (Lesueur, 1817) | |  |  |  |  |  |  |  |  |  | 1 |  |  |  |  |  |
| Cont. |  |  | Region | GA | | | | EC | | SC | | | SWC | | WC | | Gui | |
| Order | Family | Species | Depth Range | U | C | A | B | U | C | U | C | A | U | C | U | C | U | C |
|  |  | *Astrangia* sp. | |  | 1 |  |  | 1 |  | 1 |  |  |  |  |  |  | 1 |  |
|  | Schizocyathidae | *Pourtalocyathus hispidus* (Pourtalès, 1878) | |  | 1 |  |  |  | 2 |  |  |  |  |  |  |  |  |  |
|  | Stenocyathidae | *Stenocyathus vermiformis* (Pourtalès, 1868) | |  |  |  |  |  | 1 |  |  |  |  |  |  | 1 |  |  |
| Anthoathecatae | Stylasteridae | *Crypthelia glossopoma* Cairns, 1986 | |  |  |  |  |  | 2 |  |  |  |  |  |  |  |  |  |
|  |  | *Crypthelia peircei* Pourtalès, 1867 | |  | 1 |  |  |  |  |  |  |  |  |  |  |  |  |  |
